# The impact of surging transplantation of alcohol-associated liver disease on transplantation for HCC and other indications

**DOI:** 10.1097/HC9.0000000000000455

**Published:** 2024-07-05

**Authors:** Divya Ayyala-Somayajula, Jennifer L. Dodge, Kali Zhou, Norah A. Terrault, Liyun Yuan

**Affiliations:** 1 Division of Gastrointestinal and Liver Disease, Department of Medicine, Keck School of Medicine, University of Southern California, Los Angeles, California, USA; 2 Department of Population and Public Health Sciences, University of Southern California, Los Angeles, California, USA

## Abstract

**Background:**

Liver transplantation (LT) for alcohol-associated liver disease (ALD) is increasing and may impact LT outcomes for patients listed for HCC and other indications.

**Methods:**

Using US adults listed for primary LT (grouped as ALD, HCC, and other) from October 8, 2015, to December 31, 2021, we examined the impact of center-level ALD LT volume (ATxV) on waitlist outcomes in 2 eras: Era 1 (6-month wait for HCC) and Era 2 (MMaT-3). The tertile distribution of ATxV (low to high) was derived from the listed candidates as Tertile 1 (T1): <28.4%, Tertile 2 (T2): 28.4%–37.6%, and Tertile 3 (T3): >37.6% ALD LTs per year. Cumulative incidence of waitlist death and LT within 18 months from listing by LT indication were compared using the Gray test, stratified on eras and ATxV tertiles. Multivariable competing risk regression estimated the adjusted subhazard ratios (sHRs) for the risk of waitlist mortality and LT with interaction effects of ATxV by LT indication (interaction *p*).

**Results:**

Of 56,596 candidates listed, the cumulative waitlist mortality for those with HCC and other was higher and their LT probability was lower in high (T3) ATxV centers, compared to low (T1) ATxV centers in Era 2. However, compared to ALD (sHR: 0.92 [0.66–1.26]), the adjusted waitlist mortality for HCC (sHR: 1.15 [0.96–1.38], interaction *p* = 0.22) and other (sHR: 1.13 [0.87–1.46], interaction *p* = 0.16) were no different suggesting no differential impact of ATxV on the waitlist mortality. The adjusted LT probability for HCC (sHR: 0.89 [0.72–1.11], interaction *p* = 0.08) did not differ by AtxV while it was lower for other (sHR: 0.82 [0.67–1.01], interaction *p* = 0.02) compared to ALD (sHR: 1.04 [0.80–1.34]) suggesting a differential impact of ATxV on LT probability.

**Conclusions:**

The high volume of LT for ALD does not impact waitlist mortality for HCC and others but affects LT probability for other in the MMAT-3 era warranting continued monitoring.

## Graphical abstract

**Figure:**
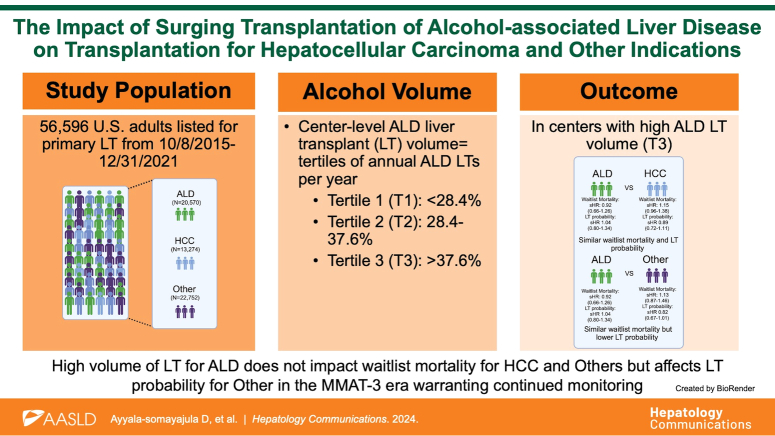

## INTRODUCTION

Equity and utility are key cornerstones of organ allocation. Before 2011, those with alcohol-associated liver disease (ALD) were disadvantaged with a lower probability of liver transplantation (LT) compared to those with other etiologies of liver disease.^1^,^2^,^3^ In contrast, those with HCC have historically experienced higher LT rates and lower waitlist mortality compared to other etiologies of liver disease,^4^,^5^ though allocation policies for HCC have evolved to reduce this disparity over time.^6^,^7^

In 2015, a 6-month wait period was instituted allowing for observance of tumor biology to facilitate dropout of those who would have poor outcomes. This reduced disparities on a national level with continued preferential allocation in short wait-time regions.^5^,^8^,^9^ To mitigate these geographic disparities, UNOS (United Network for Organ Sharing)/Organ Procurement and Transplantation Network implemented a new policy in 2019 that capped exception points (given after a 6-month wait) for patients with HCC to a median MELD-sodium (MELD-Na) within the UNOS listing region minus 3 points (MMaT−3). This reduced regional disparities but increased waitlist mortality for patients with HCC compared to patients without HCC in long wait-time regions and those with decompensated cirrhosis resulting in overall reductions in deceased donor liver transplant for HCC throughout the United States.^6^,^7^,^10^,^11^ In February 2020, donor service areas (DSAs) were changed to acuity circles within 250 nautical miles of transplant centers resulting in more equitable distribution of deceased donor liver transplant for HCC regionally.^12^

That said, the landscape of LT is very dynamic, and new or changing indications for LT can have a significant effect on access to LT and waitlist mortality for others on the list. The relatively new indication for LT, alcohol-associated hepatitis, and the removal of 6-month sobriety requirements in many states has led to a rapid rise in LT for ALD.^13^,^14^,^15^ ALD is now the leading indication for LT, accounting for ~40% of LT waitlist additions and ~50% of recipients of LT in the United States in 2021.^14^,^16^,^17^,^18^,^19^

Studies have shown that the various policies instituted for HCC have helped to reduce disparities in waitlist outcomes for those with HCC and those with other indications for LT^6^,^7^,^8^,^9^. However, in a rapidly evolving transplant landscape with the exponential rise in LT for ALD, there is concern that an unintentional disadvantage might occur for other indications for LT, including HCC.^20^,^21^ This rise in ALD as an indication for LT also coincided with MMaT−3, the newest MELD exception policy for HCC, and acuity circles changing donor organ allocation. Recent studies highlight the positive effect of the MMaT−3 policy on waitlist mortality for both HCC and non-HCC indications, concluding that the policy change had reduced geographical disparities in access to LT.^6^,^7^ However, others have highlighted that increased LT rates for ALD, driven by higher MELD at listing (≥30) among those with alcohol-associated hepatitis, may inadvertently worsen disparities in waitlist outcomes for other indications.^15^,^18^ These observations form the basis for our inquiry into the simultaneous effects of increased LT for ALD and HCC policy eras on transplant outcomes of HCC and others.

In this study, we sought to evaluate how high transplant volume of ALD coupled with policy changes for HCC influenced waitlist outcomes and the probability of LT.^20^,^21^ Our specific aims were (1) to characterize changes in alcohol transplant volume over 2 eras of HCC policy, (2) to examine the impact of alcohol transplant volume on waitlist mortality and achieving LT by era, and (3) to test whether alcohol transplant volume differentially impacted waitlist mortality and LT by indications (HCC vs. non-HCC) and eras.

## METHODS

### Data source and study population

This was a retrospective cohort study of adults ≥18 years of age listed for primary LT in the UNOS database (released March 2023) from October 8, 2015, to December 31, 2021, allowing all subjects the opportunity for 12 months follow-up through December 31, 2022. Exclusion criteria included patients listed at a center performing a lower number of LT averaging <20 per year, listed as status 1A or inactive, listed for multiorgan transplants (except kidney), listed with non-HCC exceptions, or received living donor LT.

### Transplant indication

**FIGURE 1:**
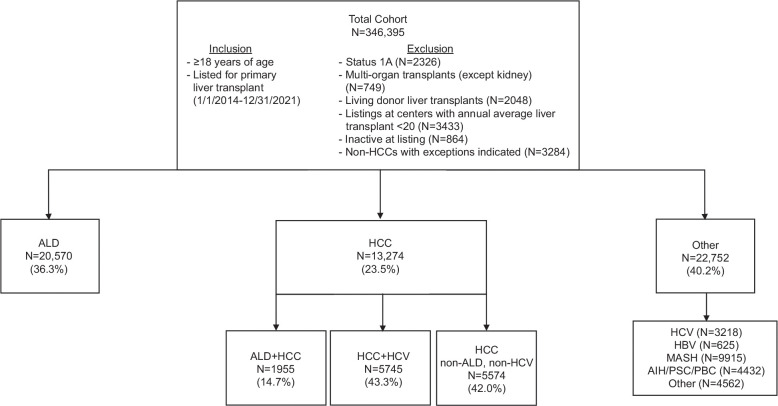
Study flowchart of the total study cohort.

Patients were grouped into 3 LT indication categories: ALD, HCC (including those with HCC + ALD), and other (all non-ALD indications without HCC) (Figure 1). HCC was defined by having an approved HCC MELD exception (including ALD + HCC). ALD included alcohol-associated cirrhosis and acute alcohol–associated hepatitis. For a subanalysis, other was further categorized as metabolic dysfunction–associated steatohepatitis (MASH), viral hepatitis, and immune-mediated liver disease. All data were publicly available and deemed exempt by the University of Southern California Institutional Review Board.

### HCC transplant policy era

As the study period spanned major changes in HCC LT policies, patients were divided into 2 cohorts reflecting the HCC policy under which they were listed: the Era 1 cohort included listings from October 8, 2015, to May 13, 2019, during the 6-month wait HCC policy and the Era 2 cohort included listings from May 14, 2019, to December 31, 2021, after the implementation of MMaT−3 policy for HCC. Although Era 2 included a change in DSA instituted on February 4, 2020, we did not separate these into 2 eras given the small number of events that occurred before the DSA changes for the main analyses.

### Transplant volume for ALD

**FIGURE 2:**
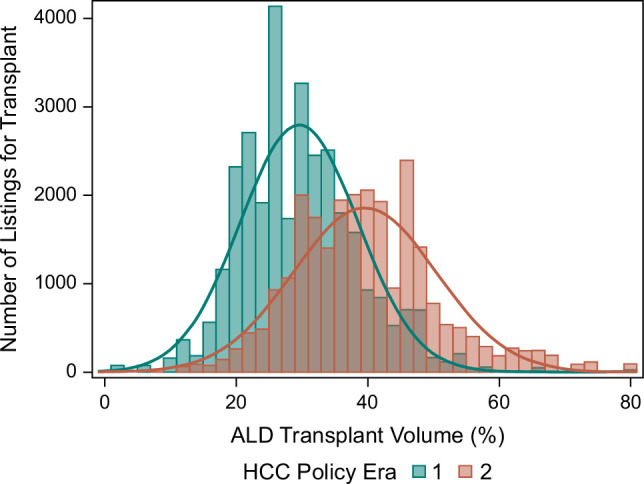
Distribution of study subjects by ALD transplant volume percent and era. Alcohol transplant volume (ATxV) was quantified as the center-level percentage of adult primary LTs for ALD (without HCC) each year (a relative proportion within a center). The number of listings by ATxV and era are shown in this figure. Abbreviations: ALD, alcohol-associated liver disease; LT, liver transplantation.

To standardize transplantation volume for ALD (ATxV) in the context of overall LT volume, ATxV was quantified as the center-level percentage of adult primary LTs for ALD (without HCC) annually (relative proportion within a center). The number of listings by ATxV and era is shown in Figure 2. Transplant candidates were assigned an ATxV tertile corresponding to their center and year of listing (Supplemental Table S1, http://links.lww.com/HC9/A926). The functional form of the association between ATxV and mortality was assessed by plotting the martingale residuals from the null proportional hazards model against alcohol transplant volume with a smoothing spline revealing a nonlinear relationship. Thus, ATxV was categorized as tertiles of increasing ALD transplant volume (Tertile 1 [T1]: <28.4%, Tertile 2 [T2]: 28.4%–37.6%, and Tertile 3 [T3]: >37.6%, ALD LTs per year) derived from the distribution of listed candidates in the study population that year. LT centers in T3 were considered high ATxV centers.

### Outcomes

The primary outcome was waitlist mortality defined as death due to any cause or dropout for clinical deterioration resulting in waitlist removal. The secondary outcome was LT from deceased donors. Patients were followed from the date of listing to the first of waitlist death, LT, waitlist removal, or 18 months of follow-up (up to January 6, 2023).

### Statistical analyses

Demographic and clinical characteristics were described by ATxV tertiles. Cumulative incidence of waitlist death and LT were estimated by ATxV tertile stratified on policy era and LT indication and compared using the Gray test. Fine and Gray competing risk regression estimated the subhazard ratios (sHRs) and 95% CIs separately for the risk of death and LT within each policy era while accounting for the competing risk (the opposing event). The multivariable models were adjusted for characteristics selected a priori including sex, race/ethnicity, public insurance, diabetes, dialysis, body mass index, Child-Pugh class, laboratory MELD at listing, region, age, and blood type. The interaction effect of ATxV tertile and LT indication was assessed to determine if there was a differential impact of ATxV on outcomes by LT indication. In a subanalysis to explore other indications for LT, the interaction between ATxV tertile and subgroups of other LT indications was tested in a multivariable model adjusted for the same covariates. In sensitivity analyses, multivariable models were executed (i) in the acuity circle era alone and (ii) using different ATxV cutoffs (quartiles and quintiles) to determine if the results were robust.

All models were assessed for multicollinearity using the variance inflation factor (all <10). Statistical analyses were performed using SAS software, version 9.4 (SAS Institute Inc.).

## RESULTS

A total of 56,596 candidates were listed, with the candidate characteristics by ATxV tertiles (T1 [N = 18,333], T2 [N = 18,588], T3 [N = 19,675]) shown in Table 1. In high ATxV centers (T3) with more than 37.6% ALD LTs per year, there were higher percentages of patients with ALD (T3: 45.5% vs. T1: 27.4%), on dialysis (T3: 12.0% vs. T1: 6.9%), with Child-Pugh class C (T3: 51.6% vs. T1: 43.7%), and with public insurance (T3: 51.0% vs. T1: 46.6%) (Table 1).

**TABLE 1:** Characteristics of the study population by alcohol transplant volume tertile

	Alcohol transplant volume tertile			
	Total	1	2	3
	N = 56,596 (100%)	N = 18,333 (32.4%)	N = 18,588 (32.8%)	N = 19,675 (34.8%)
Median age at listing (IQR)	58 (50–64)	59 (52–64)	58 (51–64)	57 (49–64)
Male, n (%)	36,356 (64.2)	12,000 (65.5)	11,801 (63.5)	12,555 (63.8)
Ethnicity, n (%)				
White	39,758 (70.2)	13,131 (71.6)	12,915 (69.5)	13,712 (69.7)
Hispanic	9815 (17.3)	2717 (14.8)	3381 (18.2)	3717 (18.9)
Black	3932 (6.9)	1276 (7.0)	1375 (7.4)	1281 (6.5)
Asian	2236 (4.0)	882 (4.8)	687 (3.7)	667 (3.4)
Other	855 (1.5)	327 (1.8)	230 (1.2)	298 (1.5)
Etiology of liver disease, n (%)				
HCC	13,274 (23.5)	5227 (28.5)	4234 (22.8)	3813 (19.4)
ALD, non-HCC	20,570 (36.3)	5022 (27.4)	6591 (35.5)	8957 (45.5)
Other, non-HCC	22,752 (40.2)	12,855 (41.1)	9897 (39.1)	22,752 (40.2)
OPTN listing region (%)				
1	3366 (5.9)	567 (3.1)	682 (3.7)	2117 (10.8)
2	6400 (11.3)	1500 (8.2)	2019 (10.9)	2881 (14.6)
3	8057 (14.2)	2417 (13.2)	2891 (15.6)	2749 (14.0)
4	6568 (11.6)	1605 (8.8)	2639 (14.2)	2324 (11.8)
5	9203 (16.3)	2780 (15.2)	3337 (18.0)	3086 (15.7)
6	1502 (2.7)	418 (2.3)	580 (3.1)	504 (2.6)
7	4622 (8.2)	1469 (8.0)	1288 (6.9)	1865 (9.5)
8	2920 (5.2)	1272 (6.9)	663 (3.6)	985 (5.0)
9	3448 (6.1)	1794 (9.8)	774 (4.2)	880 (4.5)
10	4876 (8.6)	1403 (7.7)	2365 (12.7)	1108 (5.6)
11	5634 (10.0)	3108 (17.0)	1350 (7.3)	1176 (6.0)
Public insurance at listing, n (%)	27,512 (48.6)	8547 (46.6)	8932 (48.1)	10,033 (51.0)
Initial BMI, n (%)^a^				
≥30	23,377 (41.4)	7701 (42.1)	7658 (41.2)	8018 (40.8)
25–29.9	19,044 (33.7)	6200 (33.9)	6281 (33.8)	6563 (33.4)
18.5–24.9	13,416 (23.7)	4217 (23.0)	4407 (23.7)	4792 (24.4)
<18.5	674 (1.2)	193 (1.1)	222 (1.2)	259 (1.3)
Diabetes, n (%)	17,645 (31.2)	5868 (32.0)	5926 (31.9)	5851 (29.7)
Blood type, n (%)				
A	21,193 (37.4)	6874 (37.5)	6914 (37.2)	7405 (37.6)
AB	2315 (4.1)	711 (3.9)	772 (4.2)	832 (4.2)
B	6923 (12.2)	2234 (12.2)	2268 (12.2)	2421 (12.3)
O	26,165 (46.2)	8514 (46.4)	8634 (46.4)	9017 (45.8)
Dialysis twice weekly before listing, n (%)	5435 (9.6)	1261 (6.9)	1820 (9.8)	2354 (12.0)
Child-Pugh class at listing, n (%)				
A	9003 (15.9)	3388 (18.5)	2874 (15.5)	2741 (13.9)
B	20,697 (36.6)	6933 (37.8)	6980 (37.5)	6784 (34.5)
C	26,896 (47.5)	8012 (43.7)	8734 (47.0)	10,150 (51.6)
Median MELD at listing (IQR)^b^	18 (12–27)	17 (11–25)	18 (12–26)	20 (13–29)
Ascites at the time of listing, n (%)				
Absent	13,688 (24.2)	5322 (29.0)	4379 (23.6)	3987 (20.3)
Slight	26,370 (46.6)	8316 (45.4)	8873 (47.7)	9181 (46.7)
Moderate	16,538 (29.2)	4695 (25.6)	5336 (28.7)	6507 (33.1)
Encephalopathy at the time of listing, n (%)				
None	21,171 (37.4)	7659 (41.8)	6810 (36.6)	6702 (34.1)
1–2	30,654 (54.2)	9400 (51.3)	10,426 (56.1)	10,828 (55.0)
3–4	4771 (8.4)	1274 (6.9)	1352 (7.3)	2145 (10.9)
HCC policy era, n (%)				
6-month wait	31,293 (55.3)	14,910 (81.3)	10,938 (58.8)	5445 (27.7)
MMaT−3	25,303 (44.7)	3423 (18.7)	7650 (41.2)	14,230 (72.3)

a Missing: Total, n = 85, T1, n = 11; T2, n = 20; T3, n = 43.

Abbreviations: ALD, alcohol-associated liver disease; OPTN, Organ Procurement and Transplantation Network

### Changes in indication for LT and center-level alcohol transplant volume by era

Median follow-up was similar across eras (Era 1: 0.51 y [IQR: 0.08–1.20] and Era 2: 0.31 y [IQR: 0.04–1.03]). A greater proportion of patients in Era 2 versus Era 1 had ascites (78.0% vs. 74.1%), encephalopathy (64.7% vs. 60.9%), and were on dialysis (10.6% vs. 8.8%). The proportion classified as Child class C and median MELD increased from Era 1 (45.6% Child C/MELD 18 [IQR: 11–26]) to Era 2 (49.8% Child C/MELD 19 [IQR: 13–28]) (Supplemental Table S2, http://links.lww.com/HC9/A926).

### Cumulative incidence and predictors of waitlist mortality by alcohol transplant volume

**FIGURE 3:**
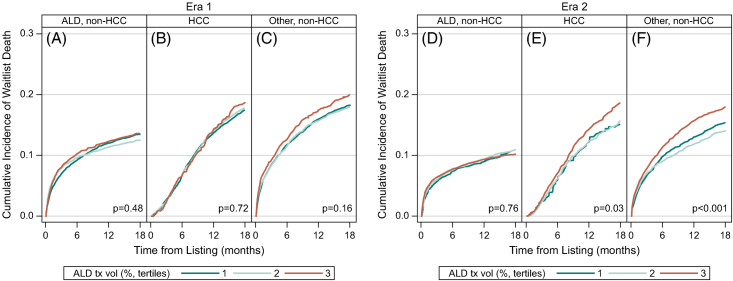
Cumulative incidence within 18 months of listing for waitlist mortality. Cumulative incidence within 18 months of listing during Era 1 for (A) ALD, (B) HCC, and (C) other and Era 2 for (D) ALD, (E) HCC, and (F) Other is shown in Figure 2. Waitlist mortality was significantly higher among those with HCC (E) and other (F) in Era 2 in the highest ATxV tertile (T3). Abbreviations: ALD, alcohol-associated liver disease; ATxV, transplant volume for alcohol-associated liver disease.

The cumulative incidence of waitlist mortality within 18 months of listing was compared by ATxV tertiles within each era and indication (Figure 3 and Supplemental Table S3, http://links.lww.com/HC9/A926). In Era 1, waitlist mortality did not significantly differ by ATxV tertile for those with ALD (*p* = 0.48), HCC (*p* = 0.72), or other indications (*p* = 0.16) (Figures 3A–C). In Era 2, waitlist mortality was significantly higher at high ATxV tertile (T3) compared to the low ATxV tertile (T1) for those with HCC (T3: 19% [17%–20%], T2: 16% [14%–18%], T1: 15% [13%–18%]) (Figure 3E, *p* = 0.03) and other indications (T3: 18% [17%–19%], T2: 14% [13%–15%], T1: 15% [14%–17%]) (Figure 3F, *p* < 0.001).

**FIGURE 4:**
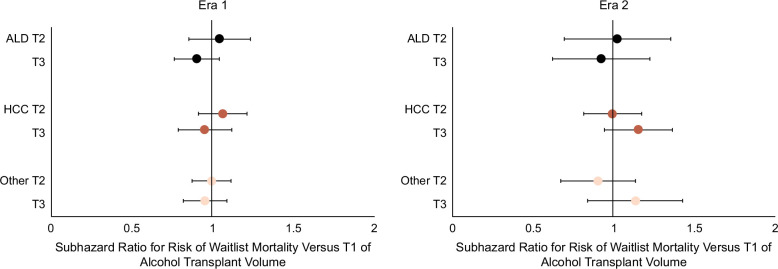
Multivariable subhazard ratios for waitlist mortality by indication and listing era. Adjusted waitlist mortality subhazard ratios for candidates listed at high (T3) versus low (T1) ATxV centers did not differ significantly among those with ALD (Era 1: 0.90 [0.77–1.04]; Era 2: ALD 0.92 [0.66–1.26]), HCC (Era 1: 0.95 [0.80–1.13], interaction *p* = 0.35; Era 2: 0.95 [0.80–1.13], interaction *p* = 0.22), and other (Era 1: 0.95 [0.82–1.09], interaction *p* = 0.33; Era 2: 1.13 [0.87–1.46], interaction *p* = 0.16) suggesting no differential effect of ATxV on waitlist mortality by indication. Multivariable models were adjusted for characteristics at listing including etiology of liver disease, sex, race/ethnicity, public insurance, diabetes, dialysis, BMI, Child-Pugh class, MELD, region, age, and blood type. Abbreviations: ALD, alcohol-associated liver disease; ATxV, transplant volume for alcohol-associated liver disease.

In multivariable-adjusted models (Figure 4 and Supplemental Table S4, http://links.lww.com/HC9/A926), waitlist mortality for candidates listed at high (T3) versus low (T1) ATxV centers did not differ significantly among those with ALD, HCC, and other indications in Era 1 (ALD: sHR 0.86 [0.72–1.04]; HCC: sHR 0.95 [0.80–1.13]; and other: sHR 0.95 [0.82–1.09]) and Era 2 (ALD: sHR 0.92 [0.66–1.26]; HCC: sHR 1.15 [0.96–1.38]; other: sHR 1.13 [0.87–1.46]). Furthermore, the effect of ATxV on waitlist mortality was similar for ALD compared to HCC and other indications (interaction: *p* = 0.22 and *p* = 0.16) (shaded Supplemental Table S4, http://links.lww.com/HC9/A926).

When exploring other indications for LT (Supplemental Table S5, http://links.lww.com/HC9/A926), the effect of high (T3) versus low (T1) ATxV center on waitlist mortality among MASH, viral hepatitis, and immune subgroups did not differ significantly from the effect among ALD in Era 1 (MASH: sHR 0.92 [0.76–1.13]; viral hepatitis: sHR 1.02 [0.78–1.35]; and immune: sHR 1.11 [0.80–1.54]). In Era 2, the risk of waitlist mortality for high (T3) versus low (T1) AtxV was higher for those with immune indications (sHR 1.57 [1.08–2.27]), differing significantly from the effect among ALD (interaction *p* = 0.001) while MASH (sHR 1.07 [0.80–1.42], interaction *p* = 0.37) and viral hepatitis (sHR 0.79 [0.54–1.14], interaction *p* = 0.48) were similar to ALD (shaded Supplemental Table S5, http://links.lww.com/HC9/A926).

### Cumulative incidence and predictors of LT by alcohol transplant volume

**FIGURE 5:**
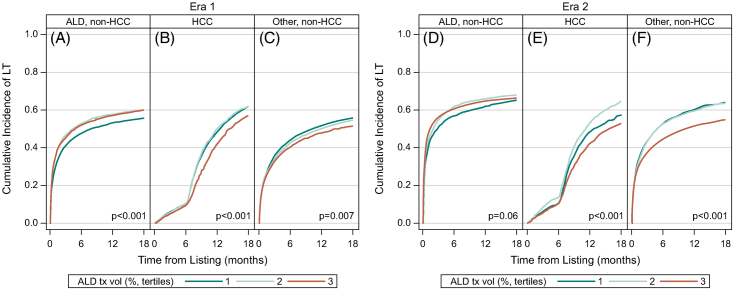
Cumulative incidence within 18 months of listing for liver transplantation. Cumulative incidence of liver transplantation within 18 months of listing during Era 1 for (A) ALD, (B) HCC, and (C) Other and Era 2 for (D) ALD, (E) HCC, and (F) other is shown in the figure. The cumulative incidence of LT was significantly lower among those with HCC (B, E) and Other (C, F) in the highest ATxV tertile (T3) in Eras 1 and 2. The cumulative incidence of LT was significantly higher among those with ALD diagnosis (A) in the highest ATxV tertiles (T2, 3) in Era 1. Abbreviations: ALD, alcohol-associated liver disease; ATxV, transplant volume for alcohol-associated liver disease; LT, liver transplantation.

The cumulative incidence of LT within 18 months of listing by indication was compared across ATxV tertiles by era (Figure 5 and Supplemental Table S3, http://links.lww.com/HC9/A926). The cumulative incidence of LT was significantly lower among those with HCC (*p* < 0.001) and other (*p* = 0.007) in the highest ATxV tertile (T3) in Era 1 (Figures 5B, C). In Era 2, the cumulative incidence of LT for those with HCC (T3: 53% [51%–55%], T2: 65% [62%–67%], T1: 57% [54%–61%], *p*=0.03) and other indications (T3: 55% [53%–56%], T2: 64% [62%–65%], T1: 64% [62%–67%], *p* < 0.001) was significantly lower in the highest ATxV tertile (T3) (Figures 5E, F, *p* < 0.001).

**FIGURE 6:**
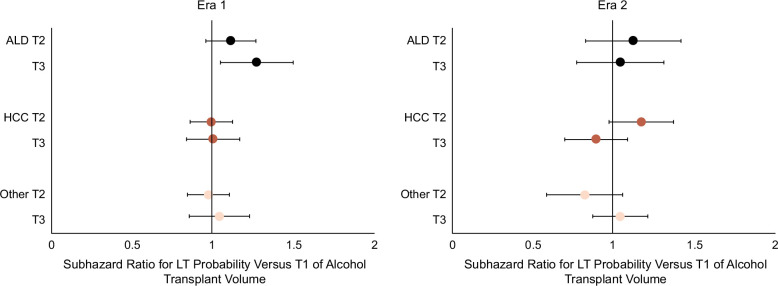
Multivariable subhazard ratios for liver transplant probability by indication and listing era. The effect of high (T3) versus low (T1) ATxV on LT differed by the etiology of liver disease. Compared to those with ALD (Era 1: 1.27 [1.07–1.52]; Era 2: ALD 1.04 [0.80–1.34]), the adjusted subhazard ratios were lower among those with HCC (Era 1: 1.00 [0.85–1.18], interaction *p* = 0.001; Era 2: 0.89 [0.72–1.11], interaction *p* = 0.08) and other (Era 1: 1.04 [0.84–1.24], interaction *p* = 0.006; Era 2: 0.82 [0.67–1.01], interaction *p* = 0.02). Multivariable models were adjusted for characteristics at listing including etiology of liver disease, sex, race/ethnicity, public insurance, diabetes, dialysis, BMI, Child-Pugh class, MELD, region, age, and blood type. Abbreviations: ALD, alcohol-associated liver disease; ATxV, transplant volume for alcohol-associated liver disease; LT, liver transplantation.

In multivariable-adjusted models (Figure 6 and Supplemental Table S6, http://links.lww.com/HC9/A926), the effect of high (T3) versus low (T1) ATxV on LT differed by etiology of liver disease. Compared to those with ALD (Era 1: 1.27 [1.07–1.52]; Era 2: ALD 1.04 [0.80–1.34]), the adjusted sHRs were lower among those with HCC (Era 1: 1.00 [0.85–1.18], interaction *p* = 0.001; Era 2: 0.89 [0.72–1.11], interaction *p* = 0.08) and other (Era 1: 1.04 [0.84–1.24], interaction *p* = 0.006; Era 2: 0.82 [0.67–1.01], interaction *p* = 0.02) suggesting a potential differential effect of TtxV on LT by indication (shaded Supplemental Table S6, http://links.lww.com/HC9/A926).

In the multivariable-adjusted models for the subgroup analysis of LT indication other (Supplemental Table S7, http://links.lww.com/HC9/A926), the effect of high (T3) versus low (T1) ATxV on LT differed by other subgroups compared to ALD with a lower adjusted sHR among those with MASH (sHR 0.81 [0.67–0.99], interaction *p*=0.01) and immune (0.70 [0.57–0.97], interaction *p*<0.001) indications, suggesting a differential effect of ATxV on LT by other indication subgroups (shaded Supplemental Table S7, http://links.lww.com/HC9/A926) in Era 2.

### Sensitivity analyses using acuity circles and different ATxV cutoffs

In a sensitivity analysis among the candidates listed for LT in the acuity circle era, the direction and magnitude of the association between ATxV and (i) waitlist mortality (Supplemental Tables S8 and S9, http://links.lww.com/HC9/A926) and (ii) probability of LT (Supplemental Tables S10 and S11, http://links.lww.com/HC9/A926) within HCC and other etiologies were relatively similar to Era 2 overall. Although interactions generally did not achieve statistical significance, the interaction between ATxV and etiology (immune vs. ALD) remained significant for the outcome of LT suggesting a differential effect of ATxV may be observed in the acuity circle era (Supplemental Table S11, http://links.lww.com/HC9/A926).

ATxV was categorized based on quartiles and quintiles to determine if the study findings were robust to different ATxV cutoffs. The results for waitlist mortality and LT were consistent across these classifications (Supplemental Tables S12–15, http://links.lww.com/HC9/A926).

## DISCUSSION

In this study examining the dual effect of increased transplantation for ALD and shifting HCC policies, we found that centers with a high volume of transplants for ALD (>37%) did not have a significantly increased risk of waitlist mortality for those with HCC and other indications compared to centers with a low volume of transplant for ALD both in the 6-month wait (Era 1) and MMaT−3 HCC policy eras (Era 2). As highlighted by other studies, we also showed that transplant centers listed more subjects with ALD as the indication for LT in current Era 2 (41.2%) compared to Era 1 (32.5%).^15^,^18^

Recent publications suggest the most recent allocation scheme (MMaT−3) has yielded an equalization of waitlist mortality rates among patients with HCC versus patients without HCC.^6^,^7^ Our study examined the influence of center-level transplant volume for ALD in the current MMaT−3 era and found a higher cumulative incidence of waitlist mortality for patients with HCC in centers with high ATxV. However, the 15% increase in waitlist mortality for patients with HCC in high ATxV centers did not reach statistical significance in multivariable models. Continued careful monitoring is warranted to determine if waitlist mortality increases under the current policy for those with HCC. If that does occur, further refinements of HCC prioritization policies may be needed. For example, consideration of starting the 6-month wait period during LT evaluation rather than at the time of listing or a stratified MELD exception point listing based on the degree of decompensation for those with HCC to mitigate possible emerging disparities for transplant. Our work highlights the importance of continual real-time assessment of the factors influencing waitlist outcomes and access to LT.

Studies have shown a reduction in LT probability for HCC after the institution of the MMaT−3 policy.^7^ We also observed a lower cumulative incidence of LT probability for HCC at high ATxV centers (T3 vs. T2 and T1) in both the 6-month wait and MMaT−3 eras. Our fully adjusted models show that the probability of LT for HCC in the MMaT−3 era was 11% lower in high ATxV centers compared to low ATxV centers. This failed to reach statistical significance suggesting no impact of high ATxV on LT probability for HCC in the MMaT−3 era during the study period. With respect to other indications, we showed that the probability of LT in the MMaT−3 era was 18% lower in high ATxV centers compared to low ATxV centers which was statistically significant (interaction *p* = 0.02) suggesting a potential adverse effect of high ATxV on LT probability for other indications in the MMaT−3 era. Furthermore, our subgroup analysis showed that this finding was likely due to a 20% and 30% lower LT probability for those listed for MASH and immune indications, respectively, in high ATxV centers compared to low ATxV centers which were both statistically significant (MASH interaction *p* = 0.02; immune interaction *p* < 0.001) in the MMaT−3 era. Although increased waitlist mortality and lower LT probability have been reported in the past for autoimmune-related liver disease, this study examines the differential effect of ATxV on waitlist outcomes for this indication and highlight a potential disparity.^22^,^23^,^24^ These findings are likely a product of an organ allocation system based on acuity which inherently favors those with underlying ALD (especially alcohol-associated hepatitis) as these patients tend to present acutely ill with higher MELD compared to their counterparts.^15^,^18^ In Era 2, we found that the number of patients listed for ALD increased and they appeared to be more decompensated (higher median MELD and increased proportion of renal insufficiency) compared to Era 1. Centers with high ATxV need to be aware of the potential impact this may have on LT probability for HCC and other indications, namely MASH and autoimmune-related liver disease. In addition, expansion of the donor pool by using living donors and marginal donors may help mitigate this disparity and ensure equitable access to LT to all indications. Finally, if this disparity coincides with increased waitlist mortality in subsequent studies, a new approach regarding adjustment of MELD exception points for HCC and possibly autoimmune-related liver disease might be required with closer consideration of the contribution of alcohol-associated hepatitis to waitlist mortality and liver transplant probability.

Our study is limited by the retrospective nature and inability to capture center-specific variation in early LT policies for ALD to further understand the impact of these policies on transplantation for patients without ALD. Moreover, the short duration between MMaT−3 and acuity circle policies resulted in few events, particularly in the HCC subgroup, limiting our ability to separate these policies for analysis. Of 6745 patients listed during MMaT−3, only 496 died (52 HCC, 187 ALD, and 257 other) before the institution of acuity circles. Thus, there may be residual confounding due to this policy change in Era 2. In addition, the effect of the COVID-19 pandemic, coinciding with Era 2, is not accounted for in this analysis though we hypothesize that lack of access to care would be an issue for patients with liver disease for all indications and are unlikely to confound our results. ALD listing and transplantation continue to rise after the pandemic highlighting the importance of continued observation of this trend.^18^ Finally, as UNOS lacks granularity in capturing the reason for waitlist removal, we cannot differentiate between HCC tumor progression and liver-related deterioration versus non-liver reasons for deterioration.

The strengths of this present study include the large sample size from a national database and the inclusion of policy eras to not only elucidate trends over time but also provide an understanding of current transplant practices on a national scale. Finally, the novel use of center-level alcohol transplant volume as a primary predictor acts as a proxy for the level of competition between etiologies for LT that is standardized across the United States to allow for an understanding of national LT trends despite the heterogeneity of early LT practices for ALD.^20^

In conclusion, our study suggests that the high volume of LT for ALD coupled with changing HCC policies has not negatively impacted waitlist mortality or LT probabilities for HCC, suggesting effective allocation policy changes. However, we observed that high-volume LT for ALD was associated with lower LT probabilities for other indications, namely MASH and autoimmune-related liver disease. Given the high variability in LT volume for ALD across the United States,^20^,^25^ continued attention and surveillance on how ALD transplant volumes may contribute to disparities in waitlist and LT outcomes for non-ALD indications is important, particularly under the recently introduced MMaT−3 policy and acuity circles.

## Supplementary Material

SUPPLEMENTARY MATERIAL
