# Supplementary material for: The impact of surging transplantation of alcohol-associated liver disease on transplantation for HCC and other indications
Source: Hepatol Commun. 2024 Jul 5;8(7):e0455. doi: 10.1097/HC9.0000000000000455 (PMC11227353; doi:10.1097/HC9.0000000000000455)
Supplement: SUPPLEMENTARY MATERIAL [file hc9-8-e0455-s001.docx]

Supplemental Table 1: Total Number of Transplants and the Number of Alcohol Associated Liver Disease (ALD) Transplants by Center and Alcohol Transplant Volume (ATxV) By Year

| 2015 | | | | 2016 | | | | 2017 | | | | 2018 | | | | 2019 | | | | 2020 | | | | 2021 | | | |
| --- | --- | --- | --- | --- | --- | --- | --- | --- | --- | --- | --- | --- | --- | --- | --- | --- | --- | --- | --- | --- | --- | --- | --- | --- | --- | --- | --- |
| **ALD LT (N)** | **Total LT (N)** | **ATxV (%)** | **ATxV Tertile** | **ALD LT (N)** | **Total LT (N)** | **ATxV (%)** | **ATxV Tertile** | **ALD LT (N)** | **Total LT (N)** | **ATxV (%)** | **ATxV Tertile** | **ALD LT (N)** | **Total LT (N)** | **ATxV (%)** | **ATxV Tertile** | **ALD LT (N)** | **Total LT (N)** | **ATxV (%)** | **ATxV Tertile** | **ALD LT (N)** | **Total LT (N)** | **ATxV (%)** | **ATxV Tertile** | **ALD LT (N)** | **Total LT (N)** | **ATxV (%)** | **ATxV Tertile** |
| 0 | 7 | 0.0 | 1 | 4 | 39 | 10.3 | 1 | 1 | 38 | 2.6 | 1 | 2 | 29 | 6.9 | 1 | 3 | 38 | 7.9 | 1 | 4 | 32 | 12.5 | 1 | 7 | 40 | 17.5 | 1 |
| 1 | 19 | 5.3 | 1 | 4 | 37 | 10.8 | 1 | 2 | 36 | 5.6 | 1 | 5 | 30 | 16.7 | 1 | 3 | 27 | 11.1 | 1 | 9 | 62 | 14.5 | 1 | 9 | 47 | 19.1 | 1 |
| 4 | 41 | 9.8 | 1 | 3 | 27 | 11.1 | 1 | 4 | 34 | 11.8 | 1 | 21 | 115 | 18.3 | 1 | 15 | 105 | 14.3 | 1 | 6 | 29 | 20.7 | 1 | 23 | 118 | 19.5 | 1 |
| 9 | 84 | 10.7 | 1 | 4 | 34 | 11.8 | 1 | 5 | 39 | 12.8 | 1 | 5 | 27 | 18.5 | 1 | 27 | 163 | 16.6 | 1 | 29 | 138 | 21.0 | 1 | 37 | 156 | 23.7 | 1 |
| 7 | 64 | 10.9 | 1 | 10 | 79 | 12.7 | 1 | 3 | 22 | 13.6 | 1 | 6 | 32 | 18.8 | 1 | 7 | 41 | 17.1 | 1 | 17 | 78 | 21.8 | 1 | 8 | 32 | 25.0 | 1 |
| 5 | 45 | 11.1 | 1 | 5 | 38 | 13.2 | 1 | 8 | 55 | 14.5 | 1 | 14 | 74 | 18.9 | 1 | 23 | 125 | 18.4 | 1 | 29 | 124 | 23.4 | 1 | 19 | 76 | 25.0 | 1 |
| 3 | 27 | 11.1 | 1 | 2 | 13 | 15.4 | 1 | 7 | 46 | 15.2 | 1 | 7 | 37 | 18.9 | 1 | 9 | 47 | 19.1 | 1 | 8 | 33 | 24.2 | 1 | 30 | 114 | 26.3 | 1 |
| 8 | 69 | 11.6 | 1 | 11 | 71 | 15.5 | 1 | 11 | 72 | 15.3 | 1 | 24 | 125 | 19.2 | 1 | 4 | 19 | 21.1 | 1 | 35 | 142 | 24.6 | 1 | 29 | 103 | 28.2 | 1 |
| 6 | 50 | 12.0 | 1 | 7 | 43 | 16.3 | 1 | 18 | 115 | 15.7 | 1 | 6 | 31 | 19.4 | 1 | 26 | 122 | 21.3 | 1 | 24 | 96 | 25.0 | 1 | 36 | 122 | 29.5 | 2 |
| 13 | 108 | 12.0 | 1 | 6 | 36 | 16.7 | 1 | 23 | 133 | 17.3 | 1 | 19 | 97 | 19.6 | 1 | 10 | 46 | 21.7 | 1 | 20 | 80 | 25.0 | 1 | 35 | 113 | 31.0 | 2 |
| 11 | 88 | 12.5 | 1 | 20 | 117 | 17.1 | 1 | 8 | 46 | 17.4 | 1 | 10 | 49 | 20.4 | 1 | 24 | 107 | 22.4 | 1 | 10 | 40 | 25.0 | 1 | 22 | 71 | 31.0 | 2 |
| 4 | 30 | 13.3 | 1 | 4 | 22 | 18.2 | 1 | 9 | 50 | 18.0 | 1 | 11 | 53 | 20.8 | 1 | 11 | 47 | 23.4 | 1 | 24 | 93 | 25.8 | 1 | 44 | 142 | 31.0 | 2 |
| 9 | 66 | 13.6 | 1 | 6 | 33 | 18.2 | 1 | 22 | 115 | 19.1 | 1 | 16 | 76 | 21.1 | 1 | 7 | 29 | 24.1 | 1 | 12 | 44 | 27.3 | 1 | 44 | 141 | 31.2 | 2 |
| 4 | 27 | 14.8 | 1 | 13 | 70 | 18.6 | 1 | 22 | 114 | 19.3 | 1 | 11 | 51 | 21.6 | 1 | 32 | 127 | 25.2 | 1 | 34 | 123 | 27.6 | 1 | 17 | 54 | 31.5 | 2 |
| 5 | 32 | 15.6 | 1 | 11 | 59 | 18.6 | 1 | 16 | 82 | 19.5 | 1 | 29 | 134 | 21.6 | 1 | 30 | 119 | 25.2 | 1 | 33 | 118 | 28.0 | 1 | 6 | 19 | 31.6 | 2 |
| 9 | 57 | 15.8 | 1 | 7 | 36 | 19.4 | 1 | 34 | 171 | 19.9 | 1 | 16 | 73 | 21.9 | 1 | 18 | 70 | 25.7 | 1 | 21 | 74 | 28.4 | 1 | 52 | 159 | 32.7 | 2 |
| 5 | 31 | 16.1 | 1 | 12 | 61 | 19.7 | 1 | 12 | 60 | 20.0 | 1 | 23 | 104 | 22.1 | 1 | 31 | 120 | 25.8 | 1 | 21 | 73 | 28.8 | 2 | 26 | 79 | 32.9 | 2 |
| 7 | 43 | 16.3 | 1 | 8 | 40 | 20.0 | 1 | 7 | 35 | 20.0 | 1 | 22 | 99 | 22.2 | 1 | 29 | 110 | 26.4 | 1 | 27 | 93 | 29.0 | 2 | 51 | 154 | 33.1 | 2 |
| 2 | 12 | 16.7 | 1 | 15 | 75 | 20.0 | 1 | 21 | 101 | 20.8 | 1 | 12 | 54 | 22.2 | 1 | 16 | 60 | 26.7 | 1 | 44 | 151 | 29.1 | 2 | 17 | 51 | 33.3 | 2 |
| 5 | 30 | 16.7 | 1 | 18 | 89 | 20.2 | 1 | 20 | 93 | 21.5 | 1 | 6 | 27 | 22.2 | 1 | 26 | 96 | 27.1 | 1 | 38 | 130 | 29.2 | 2 | 28 | 83 | 33.7 | 2 |
| 17 | 100 | 17.0 | 1 | 7 | 34 | 20.6 | 1 | 8 | 37 | 21.6 | 1 | 26 | 116 | 22.4 | 1 | 43 | 157 | 27.4 | 1 | 51 | 167 | 30.5 | 2 | 67 | 194 | 34.5 | 2 |
| 15 | 87 | 17.2 | 1 | 24 | 116 | 20.7 | 1 | 12 | 55 | 21.8 | 1 | 10 | 42 | 23.8 | 1 | 35 | 127 | 27.6 | 1 | 27 | 88 | 30.7 | 2 | 9 | 26 | 34.6 | 2 |
| 10 | 56 | 17.9 | 1 | 16 | 77 | 20.8 | 1 | 16 | 73 | 21.9 | 1 | 6 | 25 | 24.0 | 1 | 13 | 47 | 27.7 | 1 | 21 | 68 | 30.9 | 2 | 8 | 23 | 34.8 | 2 |
| 40 | 219 | 18.3 | 1 | 32 | 151 | 21.2 | 1 | 9 | 40 | 22.5 | 1 | 14 | 58 | 24.1 | 1 | 15 | 54 | 27.8 | 1 | 17 | 55 | 30.9 | 2 | 68 | 195 | 34.9 | 2 |
| 16 | 86 | 18.6 | 1 | 24 | 113 | 21.2 | 1 | 5 | 22 | 22.7 | 1 | 37 | 153 | 24.2 | 1 | 16 | 57 | 28.1 | 1 | 25 | 79 | 31.6 | 2 | 30 | 86 | 34.9 | 2 |
| 27 | 141 | 19.1 | 1 | 9 | 42 | 21.4 | 1 | 21 | 92 | 22.8 | 1 | 11 | 45 | 24.4 | 1 | 9 | 32 | 28.1 | 1 | 20 | 63 | 31.7 | 2 | 20 | 57 | 35.1 | 2 |
| 14 | 72 | 19.4 | 1 | 13 | 59 | 22.0 | 1 | 19 | 83 | 22.9 | 1 | 13 | 52 | 25.0 | 1 | 32 | 111 | 28.8 | 2 | 42 | 132 | 31.8 | 2 | 32 | 91 | 35.2 | 2 |
| 14 | 70 | 20.0 | 1 | 17 | 77 | 22.1 | 1 | 25 | 108 | 23.1 | 1 | 13 | 52 | 25.0 | 1 | 17 | 58 | 29.3 | 2 | 49 | 154 | 31.8 | 2 | 46 | 130 | 35.4 | 2 |
| 25 | 124 | 20.2 | 1 | 25 | 111 | 22.5 | 1 | 35 | 149 | 23.5 | 1 | 7 | 28 | 25.0 | 1 | 8 | 27 | 29.6 | 2 | 23 | 70 | 32.9 | 2 | 17 | 48 | 35.4 | 2 |
| 10 | 49 | 20.4 | 1 | 19 | 83 | 22.9 | 1 | 4 | 17 | 23.5 | 1 | 28 | 110 | 25.5 | 1 | 30 | 101 | 29.7 | 2 | 36 | 107 | 33.6 | 2 | 38 | 107 | 35.5 | 2 |
| 17 | 83 | 20.5 | 1 | 14 | 60 | 23.3 | 1 | 15 | 63 | 23.8 | 1 | 35 | 137 | 25.5 | 1 | 19 | 63 | 30.2 | 2 | 7 | 20 | 35.0 | 2 | 36 | 100 | 36.0 | 2 |
| 8 | 39 | 20.5 | 1 | 15 | 64 | 23.4 | 1 | 24 | 100 | 24.0 | 1 | 23 | 90 | 25.6 | 1 | 13 | 43 | 30.2 | 2 | 48 | 137 | 35.0 | 2 | 49 | 135 | 36.3 | 2 |
| 6 | 29 | 20.7 | 1 | 24 | 99 | 24.2 | 1 | 14 | 58 | 24.1 | 1 | 32 | 125 | 25.6 | 1 | 25 | 82 | 30.5 | 2 | 12 | 34 | 35.3 | 2 | 8 | 22 | 36.4 | 2 |
| 5 | 24 | 20.8 | 1 | 31 | 126 | 24.6 | 1 | 8 | 33 | 24.2 | 1 | 7 | 27 | 25.9 | 1 | 59 | 193 | 30.6 | 2 | 23 | 65 | 35.4 | 2 | 42 | 114 | 36.8 | 2 |
| 24 | 115 | 20.9 | 1 | 29 | 116 | 25.0 | 1 | 26 | 106 | 24.5 | 1 | 6 | 23 | 26.1 | 1 | 30 | 98 | 30.6 | 2 | 58 | 162 | 35.8 | 2 | 27 | 71 | 38.0 | 3 |
| 18 | 86 | 20.9 | 1 | 23 | 92 | 25.0 | 1 | 21 | 85 | 24.7 | 1 | 36 | 138 | 26.1 | 1 | 19 | 62 | 30.6 | 2 | 44 | 121 | 36.4 | 2 | 32 | 84 | 38.1 | 3 |
| 4 | 19 | 21.1 | 1 | 42 | 165 | 25.5 | 1 | 23 | 93 | 24.7 | 1 | 11 | 42 | 26.2 | 1 | 49 | 157 | 31.2 | 2 | 28 | 77 | 36.4 | 2 | 26 | 68 | 38.2 | 3 |
| 28 | 133 | 21.1 | 1 | 17 | 66 | 25.8 | 1 | 5 | 20 | 25.0 | 1 | 16 | 61 | 26.2 | 1 | 17 | 54 | 31.5 | 2 | 41 | 111 | 36.9 | 2 | 26 | 68 | 38.2 | 3 |
| 10 | 47 | 21.3 | 1 | 32 | 122 | 26.2 | 1 | 27 | 108 | 25.0 | 1 | 30 | 114 | 26.3 | 1 | 18 | 57 | 31.6 | 2 | 41 | 110 | 37.3 | 2 | 17 | 44 | 38.6 | 3 |
| 24 | 110 | 21.8 | 1 | 37 | 141 | 26.2 | 1 | 22 | 87 | 25.3 | 1 | 20 | 76 | 26.3 | 1 | 25 | 79 | 31.6 | 2 | 31 | 83 | 37.3 | 2 | 24 | 62 | 38.7 | 3 |
| 25 | 114 | 21.9 | 1 | 30 | 114 | 26.3 | 1 | 12 | 47 | 25.5 | 1 | 27 | 101 | 26.7 | 1 | 16 | 50 | 32.0 | 2 | 37 | 99 | 37.4 | 2 | 65 | 166 | 39.2 | 3 |
| 36 | 163 | 22.1 | 1 | 33 | 125 | 26.4 | 1 | 31 | 121 | 25.6 | 1 | 16 | 58 | 27.6 | 1 | 42 | 131 | 32.1 | 2 | 41 | 109 | 37.6 | 3 | 56 | 142 | 39.4 | 3 |
| 19 | 85 | 22.4 | 1 | 15 | 56 | 26.8 | 1 | 28 | 109 | 25.7 | 1 | 20 | 72 | 27.8 | 1 | 42 | 130 | 32.3 | 2 | 56 | 146 | 38.4 | 3 | 27 | 68 | 39.7 | 3 |
| 15 | 67 | 22.4 | 1 | 39 | 143 | 27.3 | 1 | 18 | 70 | 25.7 | 1 | 21 | 75 | 28.0 | 1 | 25 | 77 | 32.5 | 2 | 37 | 96 | 38.5 | 3 | 41 | 102 | 40.2 | 3 |
| 10 | 44 | 22.7 | 1 | 40 | 144 | 27.8 | 1 | 35 | 134 | 26.1 | 1 | 9 | 32 | 28.1 | 1 | 48 | 147 | 32.7 | 2 | 22 | 57 | 38.6 | 3 | 18 | 44 | 40.9 | 3 |
| 10 | 44 | 22.7 | 1 | 11 | 39 | 28.2 | 1 | 29 | 111 | 26.1 | 1 | 25 | 88 | 28.4 | 1 | 28 | 85 | 32.9 | 2 | 25 | 64 | 39.1 | 3 | 43 | 105 | 41.0 | 3 |
| 16 | 70 | 22.9 | 1 | 13 | 46 | 28.3 | 1 | 22 | 83 | 26.5 | 1 | 21 | 73 | 28.8 | 2 | 11 | 33 | 33.3 | 2 | 18 | 46 | 39.1 | 3 | 32 | 78 | 41.0 | 3 |
| 11 | 48 | 22.9 | 1 | 6 | 21 | 28.6 | 2 | 19 | 71 | 26.8 | 1 | 23 | 79 | 29.1 | 2 | 33 | 97 | 34.0 | 2 | 18 | 46 | 39.1 | 3 | 14 | 34 | 41.2 | 3 |
| 9 | 39 | 23.1 | 1 | 29 | 101 | 28.7 | 2 | 17 | 63 | 27.0 | 1 | 23 | 79 | 29.1 | 2 | 36 | 105 | 34.3 | 2 | 24 | 61 | 39.3 | 3 | 19 | 46 | 41.3 | 3 |
| 23 | 98 | 23.5 | 1 | 9 | 31 | 29.0 | 2 | 22 | 78 | 28.2 | 1 | 20 | 68 | 29.4 | 2 | 10 | 29 | 34.5 | 2 | 13 | 33 | 39.4 | 3 | 64 | 153 | 41.8 | 3 |
| 7 | 29 | 24.1 | 1 | 39 | 133 | 29.3 | 2 | 18 | 63 | 28.6 | 2 | 10 | 34 | 29.4 | 2 | 9 | 26 | 34.6 | 2 | 60 | 152 | 39.5 | 3 | 59 | 141 | 41.8 | 3 |
| 33 | 136 | 24.3 | 1 | 22 | 75 | 29.3 | 2 | 8 | 28 | 28.6 | 2 | 31 | 105 | 29.5 | 2 | 43 | 124 | 34.7 | 2 | 39 | 98 | 39.8 | 3 | 58 | 138 | 42.0 | 3 |
| 30 | 122 | 24.6 | 1 | 43 | 146 | 29.5 | 2 | 21 | 71 | 29.6 | 2 | 17 | 56 | 30.4 | 2 | 32 | 92 | 34.8 | 2 | 29 | 72 | 40.3 | 3 | 50 | 117 | 42.7 | 3 |
| 12 | 48 | 25.0 | 1 | 13 | 44 | 29.5 | 2 | 36 | 120 | 30.0 | 2 | 11 | 36 | 30.6 | 2 | 44 | 126 | 34.9 | 2 | 23 | 57 | 40.4 | 3 | 30 | 70 | 42.9 | 3 |
| 23 | 91 | 25.3 | 1 | 9 | 30 | 30.0 | 2 | 53 | 174 | 30.5 | 2 | 62 | 201 | 30.8 | 2 | 27 | 77 | 35.1 | 2 | 25 | 61 | 41.0 | 3 | 46 | 106 | 43.4 | 3 |
| 19 | 74 | 25.7 | 1 | 61 | 202 | 30.2 | 2 | 38 | 124 | 30.6 | 2 | 38 | 123 | 30.9 | 2 | 16 | 45 | 35.6 | 2 | 21 | 51 | 41.2 | 3 | 49 | 112 | 43.8 | 3 |
| 16 | 61 | 26.2 | 1 | 11 | 36 | 30.6 | 2 | 35 | 114 | 30.7 | 2 | 9 | 29 | 31.0 | 2 | 30 | 84 | 35.7 | 2 | 7 | 17 | 41.2 | 3 | 62 | 140 | 44.3 | 3 |
| 14 | 53 | 26.4 | 1 | 31 | 101 | 30.7 | 2 | 33 | 107 | 30.8 | 2 | 9 | 29 | 31.0 | 2 | 10 | 28 | 35.7 | 2 | 37 | 89 | 41.6 | 3 | 12 | 27 | 44.4 | 3 |
| 14 | 52 | 26.9 | 1 | 19 | 61 | 31.1 | 2 | 32 | 102 | 31.4 | 2 | 43 | 138 | 31.2 | 2 | 27 | 75 | 36.0 | 2 | 40 | 96 | 41.7 | 3 | 5 | 11 | 45.5 | 3 |
| 16 | 59 | 27.1 | 1 | 21 | 67 | 31.3 | 2 | 27 | 86 | 31.4 | 2 | 16 | 51 | 31.4 | 2 | 35 | 94 | 37.2 | 2 | 19 | 45 | 42.2 | 3 | 27 | 59 | 45.8 | 3 |
| 6 | 22 | 27.3 | 1 | 17 | 54 | 31.5 | 2 | 8 | 25 | 32.0 | 2 | 30 | 95 | 31.6 | 2 | 36 | 95 | 37.9 | 3 | 14 | 33 | 42.4 | 3 | 81 | 177 | 45.8 | 3 |
| 7 | 25 | 28.0 | 1 | 28 | 88 | 31.8 | 2 | 28 | 87 | 32.2 | 2 | 36 | 112 | 32.1 | 2 | 55 | 144 | 38.2 | 3 | 6 | 14 | 42.9 | 3 | 33 | 72 | 45.8 | 3 |
| 14 | 50 | 28.0 | 1 | 21 | 66 | 31.8 | 2 | 10 | 31 | 32.3 | 2 | 36 | 112 | 32.1 | 2 | 58 | 150 | 38.7 | 3 | 55 | 128 | 43.0 | 3 | 39 | 85 | 45.9 | 3 |
| 15 | 53 | 28.3 | 1 | 34 | 106 | 32.1 | 2 | 10 | 31 | 32.3 | 2 | 28 | 87 | 32.2 | 2 | 31 | 80 | 38.8 | 3 | 32 | 74 | 43.2 | 3 | 13 | 28 | 46.4 | 3 |
| 17 | 60 | 28.3 | 1 | 10 | 31 | 32.3 | 2 | 10 | 30 | 33.3 | 2 | 43 | 132 | 32.6 | 2 | 50 | 129 | 38.8 | 3 | 68 | 153 | 44.4 | 3 | 36 | 77 | 46.8 | 3 |
| 27 | 95 | 28.4 | 2 | 17 | 52 | 32.7 | 2 | 39 | 117 | 33.3 | 2 | 40 | 121 | 33.1 | 2 | 21 | 54 | 38.9 | 3 | 73 | 162 | 45.1 | 3 | 9 | 19 | 47.4 | 3 |
| 12 | 42 | 28.6 | 2 | 7 | 21 | 33.3 | 2 | 13 | 39 | 33.3 | 2 | 34 | 101 | 33.7 | 2 | 31 | 79 | 39.2 | 3 | 46 | 102 | 45.1 | 3 | 49 | 102 | 48.0 | 3 |
| 6 | 21 | 28.6 | 2 | 48 | 144 | 33.3 | 2 | 31 | 92 | 33.7 | 2 | 32 | 95 | 33.7 | 2 | 30 | 76 | 39.5 | 3 | 14 | 31 | 45.2 | 3 | 62 | 128 | 48.4 | 3 |
| 15 | 52 | 28.8 | 2 | 28 | 83 | 33.7 | 2 | 14 | 41 | 34.1 | 2 | 49 | 143 | 34.3 | 2 | 33 | 83 | 39.8 | 3 | 38 | 84 | 45.2 | 3 | 53 | 109 | 48.6 | 3 |
| 9 | 31 | 29.0 | 2 | 21 | 62 | 33.9 | 2 | 33 | 95 | 34.7 | 2 | 14 | 40 | 35.0 | 2 | 20 | 50 | 40.0 | 3 | 67 | 148 | 45.3 | 3 | 20 | 41 | 48.8 | 3 |
| 39 | 132 | 29.5 | 2 | 23 | 66 | 34.8 | 2 | 8 | 23 | 34.8 | 2 | 18 | 50 | 36.0 | 2 | 16 | 40 | 40.0 | 3 | 10 | 22 | 45.5 | 3 | 26 | 53 | 49.1 | 3 |
| 25 | 84 | 29.8 | 2 | 11 | 31 | 35.5 | 2 | 31 | 89 | 34.8 | 2 | 38 | 104 | 36.5 | 2 | 13 | 32 | 40.6 | 3 | 36 | 79 | 45.6 | 3 | 78 | 157 | 49.7 | 3 |
| 33 | 108 | 30.6 | 2 | 22 | 62 | 35.5 | 2 | 22 | 63 | 34.9 | 2 | 34 | 93 | 36.6 | 2 | 56 | 134 | 41.8 | 3 | 52 | 114 | 45.6 | 3 | 36 | 72 | 50.0 | 3 |
| 8 | 26 | 30.8 | 2 | 15 | 42 | 35.7 | 2 | 39 | 110 | 35.5 | 2 | 28 | 76 | 36.8 | 2 | 23 | 55 | 41.8 | 3 | 43 | 94 | 45.7 | 3 | 8 | 16 | 50.0 | 3 |
| 41 | 133 | 30.8 | 2 | 59 | 165 | 35.8 | 2 | 56 | 155 | 36.1 | 2 | 35 | 95 | 36.8 | 2 | 56 | 133 | 42.1 | 3 | 31 | 66 | 47.0 | 3 | 14 | 28 | 50.0 | 3 |
| 10 | 32 | 31.3 | 2 | 40 | 109 | 36.7 | 2 | 12 | 33 | 36.4 | 2 | 14 | 38 | 36.8 | 2 | 48 | 113 | 42.5 | 3 | 16 | 34 | 47.1 | 3 | 32 | 63 | 50.8 | 3 |
| 38 | 119 | 31.9 | 2 | 24 | 65 | 36.9 | 2 | 27 | 73 | 37.0 | 2 | 27 | 72 | 37.5 | 2 | 54 | 127 | 42.5 | 3 | 72 | 153 | 47.1 | 3 | 29 | 57 | 50.9 | 3 |
| 9 | 28 | 32.1 | 2 | 12 | 32 | 37.5 | 2 | 13 | 35 | 37.1 | 2 | 16 | 42 | 38.1 | 3 | 40 | 94 | 42.6 | 3 | 62 | 131 | 47.3 | 3 | 75 | 147 | 51.0 | 3 |
| 7 | 21 | 33.3 | 2 | 24 | 64 | 37.5 | 2 | 9 | 24 | 37.5 | 2 | 35 | 90 | 38.9 | 3 | 19 | 44 | 43.2 | 3 | 52 | 107 | 48.6 | 3 | 44 | 84 | 52.4 | 3 |
| 29 | 84 | 34.5 | 2 | 28 | 74 | 37.8 | 3 | 12 | 32 | 37.5 | 2 | 42 | 107 | 39.3 | 3 | 26 | 60 | 43.3 | 3 | 20 | 41 | 48.8 | 3 | 19 | 36 | 52.8 | 3 |
| 18 | 52 | 34.6 | 2 | 10 | 26 | 38.5 | 3 | 35 | 93 | 37.6 | 3 | 33 | 82 | 40.2 | 3 | 53 | 119 | 44.5 | 3 | 40 | 82 | 48.8 | 3 | 51 | 96 | 53.1 | 3 |
| 49 | 141 | 34.8 | 2 | 14 | 35 | 40.0 | 3 | 16 | 42 | 38.1 | 3 | 11 | 27 | 40.7 | 3 | 9 | 20 | 45.0 | 3 | 41 | 82 | 50.0 | 3 | 47 | 88 | 53.4 | 3 |
| 7 | 20 | 35.0 | 2 | 8 | 20 | 40.0 | 3 | 15 | 39 | 38.5 | 3 | 11 | 27 | 40.7 | 3 | 50 | 110 | 45.5 | 3 | 19 | 37 | 51.4 | 3 | 53 | 98 | 54.1 | 3 |
| 20 | 54 | 37.0 | 2 | 12 | 30 | 40.0 | 3 | 10 | 26 | 38.5 | 3 | 45 | 107 | 42.1 | 3 | 42 | 92 | 45.7 | 3 | 15 | 29 | 51.7 | 3 | 58 | 105 | 55.2 | 3 |
| 17 | 44 | 38.6 | 3 | 8 | 20 | 40.0 | 3 | 52 | 134 | 38.8 | 3 | 9 | 21 | 42.9 | 3 | 48 | 104 | 46.2 | 3 | 45 | 86 | 52.3 | 3 | 15 | 27 | 55.6 | 3 |
| 21 | 53 | 39.6 | 3 | 41 | 99 | 41.4 | 3 | 11 | 28 | 39.3 | 3 | 26 | 60 | 43.3 | 3 | 65 | 140 | 46.4 | 3 | 15 | 28 | 53.6 | 3 | 88 | 152 | 57.9 | 3 |
| 36 | 90 | 40.0 | 3 | 10 | 24 | 41.7 | 3 | 23 | 57 | 40.4 | 3 | 17 | 38 | 44.7 | 3 | 24 | 51 | 47.1 | 3 | 13 | 24 | 54.2 | 3 | 25 | 42 | 59.5 | 3 |
| 14 | 35 | 40.0 | 3 | 8 | 19 | 42.1 | 3 | 13 | 32 | 40.6 | 3 | 52 | 115 | 45.2 | 3 | 29 | 61 | 47.5 | 3 | 51 | 92 | 55.4 | 3 | 42 | 70 | 60.0 | 3 |
| 8 | 20 | 40.0 | 3 | 20 | 47 | 42.6 | 3 | 26 | 60 | 43.3 | 3 | 19 | 42 | 45.2 | 3 | 12 | 25 | 48.0 | 3 | 23 | 40 | 57.5 | 3 | 60 | 98 | 61.2 | 3 |
| 10 | 25 | 40.0 | 3 | 26 | 61 | 42.6 | 3 | 44 | 100 | 44.0 | 3 | 36 | 79 | 45.6 | 3 | 17 | 34 | 50.0 | 3 | 14 | 24 | 58.3 | 3 | 19 | 31 | 61.3 | 3 |
| 10 | 22 | 45.5 | 3 | 29 | 66 | 43.9 | 3 | 9 | 19 | 47.4 | 3 | 26 | 57 | 45.6 | 3 | 19 | 38 | 50.0 | 3 | 40 | 64 | 62.5 | 3 | 29 | 46 | 63.0 | 3 |
| 17 | 36 | 47.2 | 3 | 29 | 63 | 46.0 | 3 | 28 | 59 | 47.5 | 3 | 18 | 38 | 47.4 | 3 | 14 | 27 | 51.9 | 3 | 50 | 79 | 63.3 | 3 | 21 | 32 | 65.6 | 3 |
| 16 | 33 | 48.5 | 3 | 12 | 26 | 46.2 | 3 | 27 | 55 | 49.1 | 3 | 31 | 65 | 47.7 | 3 | 23 | 43 | 53.5 | 3 | 13 | 20 | 65.0 | 3 | 53 | 80 | 66.3 | 3 |
| 26 | 53 | 49.1 | 3 | 21 | 43 | 48.8 | 3 | 39 | 73 | 53.4 | 3 | 31 | 64 | 48.4 | 3 | 21 | 38 | 55.3 | 3 | 28 | 41 | 68.3 | 3 | 10 | 15 | 66.7 | 3 |
| 21 | 39 | 53.8 | 3 | 20 | 38 | 52.6 | 3 | 21 | 39 | 53.8 | 3 | 19 | 39 | 48.7 | 3 | 40 | 70 | 57.1 | 3 | 48 | 67 | 71.6 | 3 | 42 | 62 | 67.7 | 3 |
|  |  |  |  | 10 | 15 | 66.7 | 3 | 4 | 5 | 80.0 | 3 | 23 | 45 | 51.1 | 3 | 31 | 53 | 58.5 | 3 | 28 | 38 | 73.7 | 3 | 45 | 56 | 80.4 | 3 |

Supplemental Table 2: Characteristics Of Study Population Stratified By Era

| **Characteristics at Listing** | **Total**  N=56,596 | **6-month Wait** (Era 1) N=31,293 | **MMaT-3** (Era 2) N=25,303 |
| --- | --- | --- | --- |
| **Age, median (IQR)** | 58 (50-64) | 58 (51-64) | 58 (49-64) |
| **Male (%)** | 36,356 (64.2) | 20,304 (64.9) | 16,052 (63.4) |
| **Ethnicity (%)** | | | |
| White | 39,758 (70.2) | 21,934 (70.1) | 17,824 (70.4) |
| Hispanic | 9,815 (17.3) | 5,254 (16.8) | 4,561 (18.0) |
| Black | 3,932 (6.9) | 2,346 (7.5) | 1,586 (6.3) |
| Asian | 2,236 (4.0) | 1,283 (4.1) | 953 (3.8) |
| Other | 855 (1.5) | 476 (1.5) | 379 (1.5) |
| **Etiology of Liver Disease (%)** | | | |
| HCC | 13,274 (23.5) | 8,281 (26.5) | 4,993 (19.7) |
| ALD | 20,570 (36.3) | 10,157 (32.5) | 10,413 (41.2) |
| Other | 22,752 (40.2) | 12,855 (41.1) | 9,897 (39.1) |
| **OPTN Region (%)** | | | |
| 1 | 3,366 (5.9) | 1,837 (5.9) | 1,529 (6.0) |
| 2 | 6,400 (11.3) | 3,722 (11.9) | 2,678 (10.6) |
| 3 | 8,057 (14.2) | 4,334 (13.8) | 3,723 (14.7) |
| 4 | 6,568 (11.6) | 3,507 (11.2) | 3,061 (12.1) |
| 5 | 9,203 (16.3) | 4,954 (15.8) | 4,249 (16.8) |
| 6 | 1,502 (2.7) | 876 (2.8) | 626 (2.5) |
| 7 | 4,622 (8.2) | 2,655 (8.5) | 1,967 (7.8) |
| 8 | 2,920 (5.2) | 1,646 (5.3) | 1,274 (5.0) |
| 9 | 3,448 (6.1) | 1,937 (6.2) | 1,511 (6.0) |
| 10 | 4,876 (8.6) | 2,743 (8.8) | 2,133 (8.4) |
| 11 | 5,634 (10.0) | 3,082 (9.8) | 2,552 (10.1) |
| **Public insurance (%)** | 27,512 (48.6) | 15,261 (48.8) | 12,251 (48.4) |
| **BMI kg/m^2^ (%)^a^** | | | |
| >=30 |  |  |  |
| 25-29.9 | 23,377 (41.4) | 12,963 (41.5) | 10,414 (41.2) |
| 18.5-24.9 | 19,044 (33.7) | 10,702 (34.2) | 8,342 (33.0) |
| <18.5 | 13,416 (23.7) | 7,240 (23.2) | 6,176 (24.4) |
| **Diabetes (%)** | 17,645 (31.2) | 9,934 (31.7) | 7,711 (30.5) |
| **Blood Type (%)** | | | |
| A | 21,193 (37.4) | 11,720 (37.5) | 9,473 (37.4) |
| AB | 2,315 (4.1) | 1,269 (4.1) | 1,046 (4.1) |
| B | 6,923 (12.2) | 3,821 (12.2) | 3,102 (12.3) |
| O | 26,165 (46.2) | 14,483 (46.3) | 11,682 (46.2) |
| **Dialysis twice weekly (%)** | 5,435 (9.6) | 2,751 (8.8) | 2,684 (10.6) |
| **Child Pugh Class (%)^a^** | | | |
| A | 9,003 (15.9) | 5,342 (17.1) | 3,661 (14.5) |
| B | 20,697 (36.6) | 11,667 (37.3) | 9,030 (35.7) |
| C | 26,896 (47.5) | 14,284 (45.6) | 12,612 (49.8) |
| **MELD, median (IQR)^a^** | 18 (12-27) | 18 (11-26) | 19 (13-28) |
| **Ascites (%)** | | | |
| Absent | 13,688 (24.2) | 8,112 (25.9) | 5,576 (22.0) |
| Slight | 26,370 (46.6) | 14,554 (46.5) | 11,816 (46.7) |
| Moderate | 16,538 (29.2) | 8,627 (27.6) | 7,911 (31.3) |
| **Encephalopathy (%)** | | | |
| None | 21,171 (37.4) | 12,241 (39.1) | 8,930 (35.3) |
| 1-2 | 30,654 (54.2) | 16,651 (53.2) | 14,003 (55.3) |
| 3-4 | 4,771 (8.4) | 2,401 (7.7) | 2,370 (9.4) |
| **Albumin (mg/dL), median (IQR)*** | 3.20 (2.80-3.70) | 3.20 (2.70-3.60) | 2,370 (9.4) |
| **Follow up Time (years), median (IQR)** | 0.42 (0.06-1.11) | 0.51 (0.08-1.20) | 0.31 (0.04-1.03) |
| **ALD Transplant Volume (%, Tertiles), (%)** | | | |
| 1 | 18,333 (32.4) | 14,910 (47.6) | 3,423 (13.5) |
| 2 | 18,588 (32.8) | 10,938 (35.0) | 7,650 (30.2) |
| 3 | 19,675 (34.8) | 5,445 (17.4) | 14,230 (56.2) |

^a^Missing: BMI: Missing Total, n=85; 6 month wait, n=45; MMaT-3, n=40

Supplemental Table 3: Cumulative Incidence Of Waitlist Mortality And Liver Transplantation By Etiology, Alcohol Volume Tertile, and Era

| ***Cumulative Incidence of Waitlist Mortality*** | | | | | | | | |
| --- | --- | --- | --- | --- | --- | --- | --- | --- |
| Etiology of Liver Disease | ATxV Quartile | 6-month Wait | | | MMaT-3 | | | |
|  |  | 6 month | 12 month | 18 month | 6 month | 12 month | 18 month | |
| ALD | 1 | 0.09 (0.08-0.10) | 0.12 (0.11-0.13) | 0.14 (0.12-0.15) | 0.07 (0.06-0.09) | 0.09 (0.07-0.11) | 0.11 (0.09-0.13) | |
|  | 2 | 0.10 (0.09-0.11) | 0.11 (0.10-0.12) | 0.13 (0.12-0.14) | 0.08 (0.07-0.09) | 0.09 (0.08-0.11) | 0.11 (0.10-0.12) | |
|  | 3 | 0.10 (0.09-0.12) | 0.12 (0.11-0.14) | 0.14 (0.12-0.15) | 0.08 (0.07-0.08) | 0.09 (0.09-0.10) | 0.10 (0.10-0.11) | |
| HCC | 1 | 0.07 (0.06-0.08) | 0.14 (0.13-0.15) | 0.17 (0.16-0.19) | 0.06 (0.05-0.08) | 0.12 (0.10-0.15) | 0.15 (0.13-0.18) | |
|  | 2 | 0.07 (0.06-0.08) | 0.14 (0.13-0.16) | 0.18 (0.16-0.19) | 0.06 (0.05-0.07) | 0.12 (0.01-0.14) | 0.16 (0.14-0.18) | |
|  | 3 | 0.07 (0.05-0.08) | 0.14 (0.12-0.16) | 0.19 (0.17-0.21) | 0.07 (0.06-0.08) | 0.14 (0.13-0.16) | 0.19 (0.17-0.20) | |
| Other | 1 | 0.12 (0.11-0.13) | 0.17 (0.15-0.17) | 0.18 (0.17-0.19) | 0.10 (0.08-0.11) | 0.13 (0.11-0.15) | 0.15 (0.14-0.17) | |
|  | 2 | 0.12 (0.11-0.13) | 0.16 (0.15-0.17) | 0.18 (0.17-0.19) | 0.09 (0.08-0.10) | 0.12 (0.11-0.13) | 0.14 (0.13-0.15) | |
|  | 3 | 0.13 (0.11-0.14) | 0.17 (0.16-0.19) | 0.20 (0.18-0.22) | 0.11 (0.10-0.12) | 0.16 (0.15-0.17) | 0.18 (0.17-0.19) | |
| ***Cumulative Incidence of Liver Transplant*** | | | | | | | | |
| Etiology Liver Disease | ATxV Quartile | 6-month Wait | | | MMaT-3 | | | |
|  |  | 6 month | 12 month | 18 month | 6 month | 12 month | | 18 month |
| ALD | 1 | 0.48 (0.46-0.49) | 0.53 (0.52-0.55) | 0.55 (0.54-0.57) | 0.57 (0.54-0.60) | 0.62 (0.59-0.65) | | 0.65 (0.62-0.68) |
|  | 2 | 0.53 (0.51-0.54) | 0.58 (0.56-0.59) | 0.57 (0.55-0.59) | 0.62 (0.60-0.64) | 0.66 (0.64-0.68) | | 0.68 (0.66-0.70) |
|  | 3 | 0.52 (0.50-0.54) | 0.57 (0.55-0.59) | 0.60 (0.58-0.62) | 0.61 (0.59-0.62) | 0.65 (0.63-0.66) | | 0.66 (0.65-0.67) |
| HCC | 1 | 0.10 (0.10-0.11) | 0.48 (0.47-0.50) | 0.62 (0.60-0.63) | 0.11 (0.09-0.13) | 0.48 (0.45-0.52) | | 0.57 (0.54-0.61) |
|  | 2 | 0.11 (0.09-0.12) | 0.50 (0.48-0.52) | 0.62 (0.60-0.64) | 0.14 (0.12-0.16) | 0.53 (0.51-0.56) | | 0.65 (0.62-0.67) |
|  | 3 | 0.09 (0.08-0.11) | 0.42 (0.39-0.45) | 0.57 (0.54-0.60) | 0.10 (0.09-0.12) | 0.42 (0.40-0.44) | | 0.53 (0.51-0.55) |
| Other | 1 | 0.44 (0.43-0.46) | 0.52 (0.51-0.53) | 0.56 (0.55-0.57) | 0.53 (0.50-0.55) | 0.60 (0.58-0.63) | | 0.64 (0.62-0.67) |
|  | 2 | 0.43 (0.41-0.44) | 0.50 (0.49-0.52) | 0.55 (0.53-0.56) | 0.53 (0.51-0.54) | 0.60 (0.58-0.61) | | 0.64 (0.62-0.65) |
|  | 3 | 0.40 (0.38-0.43) | 0.48 (0.46-0.50) | 0.51 (0.49-0.54) | 0.45 (0.43-0.46) | 0.52 (0.50-0.53) | | 0.55 (0.53-0.56) |

Supplementary Table 4: Multivariable^a^ Sub-Hazard Ratios For Waitlist Mortality By Indication And Listing Era

| **ATxV Tertile** | **ALD** | | **HCC** | | | **Other** | | |
| --- | --- | --- | --- | --- | --- | --- | --- | --- |
|  | **sHR^b^** | **95% CI** | **sHR^b^** | **95% CI** | **P value** | **sHR^b^** | **95% CI** | **P value** |
|  |  |  |  |  | **vs ALD^c^** |  |  | **vs ALD^c^** |
| *Era 1* | | | | | | | | |
| 1 | 1 | ref | 1 | ref | ref | 1 | ref | ref |
| 2 | 0.90 | 0.77-1.05 | 1.06 | 0.92-1.22 | 0.07 | 0.99 | 0.88-1.12 | 0.15 |
| 3 | 0.86 | 0.72-1.04 | 0.95 | 0.80-1.13 | 0.35 | 0.95 | 0.82-1.09 | 0.33 |
| *Era 2* | | | | | | | | |
| 1 | 1 | ref | 1 | ref | ref | 1 | ref | ref |
| 2 | 1.02 | 0.74-1.40 | 0.99 | 0.83-1.19 | 0.91 | 0.90 | 0.70-1.16 | 0.42 |
| 3 | 0.92 | 0.66-1.26 | 1.15 | 0.96-1.38 | 0.22 | 1.13 | 0.87-1.46 | 0.16 |

^a^ Multivariable models adjusted for characteristics at listing including etiology of liver disease, sex, race/ethnicity, public insurance, diabetes, dialysis, BMI, Child Pugh class, MELD, region, age, and blood type. **^b^** Effect sizes are sub-hazard ratios. **^c^** Etiology by ATxV interaction p-values (vs ALD)

Supplementary Table 5: Multivariable^a^ sub-Hazard Ratios for Waitlist Mortality by Other Indication Type and Listing Era

| **ATxV Tertile** | **MASH^d^** | | | **Viral hepatitis** | | | **Immune** | | |
| --- | --- | --- | --- | --- | --- | --- | --- | --- | --- |
|  | **sHR^b^** | **95% CI** | **P value**  **vs ALD^c^** | **sHR^b^** | **95% CI** | **P value** | **sHR^b^** | **95% CI** | **P value** |
|  |  |  |  |  |  | **vs ALD^c^** |  |  | **vs ALD^c^** |
| *Era 1* | | | | | | | | | |
| 1 | 1 | ref | ref | 1 | ref | ref | 1 | ref | ref |
| 2 | 1.03 | 0.86-1.23 | 0.20 | 0.97 | 0.76-1.26 | 0.44 | 0.92 | 0.73-1.17 | 0.83 |
| 3 | 0.92 | 0.76-1.13 | 0.58 | 1.02 | 0.78-1.35 | 0.22 | 1.11 | 0.80-1.54 | 0.15 |
| *Era 2* | | | | | | | | | |
| 1 | 1 | ref | ref | 1 | ref | ref | 1 | ref | ref |
| 2 | 0.79 | 0.60-1.05 | 0.15 | 0.66 | 0.45-0.96 | 0.053 | 1.26 | 0.82-1.94 | 0.27 |
| 3 | 1.07 | 0.80-1.42 | 0.37 | 0.79 | 0.54-1.14 | 0.48 | 1.57 | 1.08-2.27 | **0.001** |

^a^ Multivariable models adjusted for characteristics at listing including etiology of liver disease, sex, race/ethnicity, public insurance, diabetes, dialysis, BMI, Child Pugh class, MELD, region, age, and blood type. **^b^** Effect sizes are sub-hazard ratios. **^c^** Etiology by ATxV interaction p-values (vs ALD) ^d^ Metabolic steatohepatitis

Supplementary Table 6: Multivariable^a^ Sub-Hazard Ratios For Probability Of Liver Transplantation By Etiology And Listing Era

| **ATxV Tertile** | **ALD** | | **HCC** | | | **Other** | | |
| --- | --- | --- | --- | --- | --- | --- | --- | --- |
|  | **sHR^b^** | **95% CI** | **sHR^b^** | **95% CI** | **P value** | **sHR^b^** | **95% CI** | **P value** |
|  |  |  |  |  | **vs ALD^c^** |  |  | **vs ALD^c^** |
| *Era 1* | | | | | | | | |
| 1 | 1 | ref | 1 | ref | ref | 1 | ref | ref |
| 2 | 1.11 | 0.97-1.28 | 0.99 | 0.87-1.13 | 0.06 | 0.97 | 0.85-1.11 | **0.003** |
| 3 | **1.27** | **1.07-1.52** | 1.00 | 0.85-1.18 | **0.001** | 1.04 | 0.87-1.24 | **0.006** |
| *Era 2* | | | | | | | | |
| 1 | 1 | ref | 1 | ref | ref | 1 | ref | ref |
| 2 | 1.12 | 0.86-1.45 | 1.17 | 0.99-1.39 | 0.59 | 1.04 | 0.83-1.30 | 0.49 |
| 3 | 1.04 | 0.80-1.34 | 0.89 | 0.72-1.11 | 0.08 | 0.82 | 0.67-1.01 | **0.02** |

^a^ Multivariable models adjusted for characteristics at listing including etiology of liver disease, sex, race/ethnicity, public insurance, diabetes, dialysis, BMI, Child Pugh class, MELD, region, age, and blood type. **^b^** Effect sizes are sub-hazard ratios. **^c^** Etiology by ATxV interaction p-values (vs ALD)

Supplementary Table 7: Multivariable^a^ sub-Hazard Ratios for Probability of Liver Transplantation by Other Indication Type and Listing Era

| **ATxV Tertile** | **MASH^d^** | | | **Viral hepatitis** | | | **Immune** | | |
| --- | --- | --- | --- | --- | --- | --- | --- | --- | --- |
|  | **sHR^b^** | **95% CI** | **P value**  **vs ALD^c^** | **sHR^b^** | **95% CI** | **P value** | **sHR^b^** | **95% CI** | **P value** |
|  |  |  |  |  |  | **vs ALD^c^** |  |  | **vs ALD^c^** |
| *Era 1* | | | | | | | | | |
| 1 | 1 | ref | ref | 1 | ref | ref | 1 | ref | ref |
| 2 | 0.93 | 0.81-1.07 | **0.005** | 1.01 | 0.80-1.27 | 0.26 | 1.04 | 0.85-1.26 | 0.45 |
| 3 | 1.05 | 0.86-1.28 | **0.048** | 1.00 | 0.76-1.32 | **0.04** | 1.08 | 0.82-1.43 | 0.22 |
| *Era 2* | | | | | | | | | |
| 1 | 1 | ref | ref | 1 | ref | ref | 1 | ref | ref |
| 2 | 1.03 | 0.84-1.27 | 0.42 | 1.23 | 0.83-1.83 | 0.64 | 0.91 | 0.70-1.19 | 0.07 |
| 3 | 0.81 | 0.67-0.99 | **0.01** | 0.97 | 0.68-1.39 | 0.71 | 0.70 | 0.57-0.97 | **<0.001** |

^a^ Multivariable models adjusted for characteristics at listing including etiology of liver disease, sex, race/ethnicity, public insurance, diabetes, dialysis, BMI, Child Pugh class, MELD, region, age, and blood type. **^b^** Effect sizes are sub-hazard ratios. **^c^** Etiology by ATxV interaction p-values (vs ALD) ^d^ Metabolic steatohepatitis

Supplementary Table 8: Multivariable^a^ Sub-Hazard Ratios For Waitlist Mortality By Indication in Acuity Circle Era

| **ATxV Tertile** | **ALD** | | **HCC** | | | **Other** | | |
| --- | --- | --- | --- | --- | --- | --- | --- | --- |
|  | **sHR^b^** | **95% CI** | **sHR^b^** | **95% CI** | **P value** | **sHR^b^** | **95% CI** | **P value** |
|  |  |  |  |  | **vs ALD^c^** |  |  | **vs ALD^c^** |
| Era: Acuity circle | | | | | | | | |
| 1 | 1 | ref | 1 | ref | ref | 1 | ref | ref |
| 2 | 1.20 | 0.69-2.08 | 0.87 | 0.69-1.11 | 0.28 | 0.98 | 0.68-1.42 | 0.49 |
| 3 | 1.14 | 0.68-1.93 | 1.02 | 0.81-1.28 | 0.66 | 1.21 | 0.83-1.76 | 0.85 |

^a^ Multivariable models adjusted for characteristics at listing including etiology of liver disease, sex, race/ethnicity, public insurance, diabetes, dialysis, BMI, Child Pugh class, MELD, region, age, and blood type. **^b^** Effect sizes are sub-hazard ratios. **^c^** Etiology by ATxV interaction p-values (vs ALD)

Supplementary Table 9: Multivariable^a^ sub-Hazard Ratios for Waitlist Mortality by Other Indication Type and in Acuity Circle Era

| **ATxV Tertile** | **MASH^d^** | | | **Viral hepatitis** | | | **Immune** | | |
| --- | --- | --- | --- | --- | --- | --- | --- | --- | --- |
|  | **sHR^b^** | **95% CI** | **P value**  **vs ALD^c^** | **sHR^b^** | **95% CI** | **P value** | **sHR^b^** | **95% CI** | **P value** |
|  |  |  |  |  |  | **vs ALD^c^** |  |  | **vs ALD^c^** |
| Era: Acuity circle | | | | | | | | | |
| 1 | 1 | ref | ref | 1 | ref | ref | 1 | ref | ref |
| 2 | 0.87 | 0.57-1.32 | 0.32 | 0.75 | 0.45-1.27 | 0.22 | 1.58 | 0.85-2.93 | 0.43 |
| 3 | 1.03 | 0.66-1.59 | 0.72 | 1.20 | 0.74-1.95 | 0.90 | 1.93 | 1.08-3.42 | 0.11 |

^a^ Multivariable models adjusted for characteristics at listing including etiology of liver disease, sex, race/ethnicity, public insurance, diabetes, dialysis, BMI, Child Pugh class, MELD, region, age, and blood type. **^b^** Effect sizes are sub-hazard ratios. **^c^** Etiology by ATxV interaction p-values (vs ALD)

Supplementary Table 10: Multivariable^a^ Sub-Hazard Ratios For Probability Of Liver Transplantation By Indication in Acuity Circle Era

| **ATxV Tertile** | **ALD** | | **HCC** | | | **Other** | | |
| --- | --- | --- | --- | --- | --- | --- | --- | --- |
|  | **sHR^b^** | **95% CI** | **sHR^b^** | **95% CI** | **P value** | **sHR^b^** | **95% CI** | **P value** |
|  |  |  |  |  | **vs ALD^c^** |  |  | **vs ALD^c^** |
| Era: Acuity circle | | | | | | | | |
| 1 | 1 | ref | 1 | ref | ref | 1 | ref | ref |
| 2 | 1.01 | 0.72-1.40 | 1.10 | 0.88-1.38 | 0.50 | 0.95 | 0.70-1.28 | 0.70 |
| 3 | 0.91 | 0.66-1.25 | 0.81 | 0.63-1.04 | 0.30 | 0.75 | 0.57-0.98 | 0.19 |

^a^ Multivariable models adjusted for characteristics at listing including etiology of liver disease, sex, race/ethnicity, public insurance, diabetes, dialysis, BMI, Child Pugh class, MELD, region, age, and blood type. **^b^** Effect sizes are sub-hazard ratios. **^c^** Etiology by ATxV interaction p-values (vs ALD)

Supplementary Table 11: Multivariable^a^ sub-Hazard Ratios for Probability of Liver Transplantation by Other Indication Type in Acuity Circle Era

| **ATxV Tertile** | **MASH^d^** | | | **Viral hepatitis** | | | **Immune** | | |
| --- | --- | --- | --- | --- | --- | --- | --- | --- | --- |
|  | **sHR^b^** | **95% CI** | **P value**  **vs ALD^c^** | **sHR^b^** | **95% CI** | **P value** | **sHR^b^** | **95% CI** | **P value** |
|  |  |  |  |  |  | **vs ALD^c^** |  |  | **vs ALD^c^** |
| Era: Acuity circle | | | | | | | | | |
| 1 | 1 | ref | ref | 1 | ref | ref | 1 | ref | ref |
| 2 | 0.97 | 0.75-1.25 | 0.77 | 0.91 | 0.61-1.35 | 0.65 | 0.76 | 0.55-1.04 | 0.05 |
| 3 | 0.76 | 0.60-0.96 | 0.15 | 0.68 | 0.47-1.00 | 0.18 | 0.60 | 0.46-0.78 | **0.004** |

^a^ Multivariable models adjusted for characteristics at listing including etiology of liver disease, sex, race/ethnicity, public insurance, diabetes, dialysis, BMI, Child Pugh class, MELD, region, age, and blood type. **^b^** Effect sizes are sub-hazard ratios. **^c^** Etiology by ATxV interaction p-values (vs ALD) ^d^ Metabolic steatohepatitis

Supplementary Table 12: Multivariable^a^ Sub-Hazard Ratios For Waitlist Mortality By Indication And Listing Era in Quartiles and Quintiles

| **ATxV** | **ALD** | | **HCC** | | | **Other** | | |
| --- | --- | --- | --- | --- | --- | --- | --- | --- |
|  | **sHR^b^** | **95% CI** | **sHR^b^** | **95% CI** | **P value** | **sHR^b^** | **95% CI** | **P value** |
|  |  |  |  |  | **vs ALD^c^** |  |  | **vs ALD^c^** |
| **ATxV quartiles** | | | | | | | | |
| *Era 1* | | | | | | | | |
| 1 | 1 | ref | 1 | ref | ref | 1 | ref | ref |
| 2 | 0.83 | 0.70-0.99 | 1.01 | 0.87-1.16 | **0.04** | 0.89 | 0.77-1.02 | 0.51 |
| 3 | 0.82 | 0.69-0.96 | 1.04 | 0.88-1.22 | **0.01** | 1.00 | 0.87-1.16 | **0.01** |
| 4 | 0.88 | 0.72-1.09 | 0.91 | 0.75-1.11 | 0.82 | 0.90 | 0.75-1.08 | 0.86 |
| *Era 2* | | | | | | | | |
| 1 | 1 | ref | 1 | ref | ref | 1 | ref | ref |
| 2 | 0.97 | 0.72-1.29 | 0.91 | 0.74-1.13 | 0.77 | 0.84 | 0.64-1.09 | 0.46 |
| 3 | 0.78 | 0.54-1.11 | 0.88 | 0.70-1.11 | 0.54 | 0.83 | 0.63-1.10 | 0.69 |
| 4 | 0.77 | 0.55-1.08 | 0.95 | 0.76-1.19 | 0.28 | 1.01 | 0.76-1.35 | 0.11 |
| **ATxV quintiles** | | | | | | | | |
| *Era 1* | | | | | | | | |
| 1 | 1 | ref | 1 | ref | ref | 1 | ref | ref |
| 2 | 0.81 | 0.68-0.96 | 1.09 | 0.96-1.24 | **<0.001** | 0.92 | 0.80-1.05 | 0.18 |
| 3 | 0.79 | 0.65-0.97 | 1.09 | 0.93-1.28 | **0.005** | 0.94 | 0.81-1.08 | 0.09 |
| 4 | 0.76 | 0.62-0.93 | 1.01 | 0.85-1.20 | **0.01** | 1.00 | 0.84-1.20 | **0.01** |
| 5 | 0.84 | 0.68-1.04 | 0.85 | 0.67-1.08 | 0.96 | 0.84 | 0.68-1.04 | 0.99 |
| *Era 2* | | | | | | | | |
| 1 | 1 | ref | 1 | ref | ref | 1 | ref | ref |
| 2 | 0.76 | 0.54-1.09 | 0.76 | 0.60-0.95 | 0.96 | 0.90 | 0.62-1.32 | 0.46 |
| 3 | 0.72 | 0.50-1.03 | 0.75 | 0.58-0.97 | 0.86 | 0.77 | 0.54-1.12 | 0.70 |
| 4 | 0.65 | 0.46-0.93 | 0.88 | 0.71-1.10 | 0.16 | 0.99 | 0.69-1.42 | **0.04** |
| 5 | 0.68 | 0.48-0.97 | 0.83 | 0.65-1.06 | 0.36 | 0.98 | 0.69-1.40 | **0.04** |

^a^ Multivariable models adjusted for characteristics at listing including etiology of liver disease, sex, race/ethnicity, public insurance, diabetes, dialysis, BMI, Child Pugh class, MELD, region, age, and blood type. **^b^** Effect sizes are sub-hazard ratios. **^c^** Etiology by ATxV interaction p-values (vs ALD) ^d^ Metabolic steatohepatitis

Supplementary Table 13: Multivariable^a^ Sub-Hazard Ratios For Waitlist Mortality By Other Indication Type And Listing Era in Quartiles and Quintiles

| **ATxV** | **MASH^d^** | | | **Viral hepatitis** | | | **Immune** | | |
| --- | --- | --- | --- | --- | --- | --- | --- | --- | --- |
|  | **sHR^b^** | **95% CI** | **P value**  **vs ALD^c^** | **sHR^b^** | **95% CI** | **P value** | **sHR^b^** | **95% CI** | **P value** |
|  |  |  |  |  |  | **vs ALD^c^** |  |  | **vs ALD^c^** |
| **ATxV Quartiles** | | | | | | | | | |
| *Era 1* | | | | | | | | | |
| 1 | 1 | ref | ref | 1 | ref | ref | 1 | ref | ref |
| 2 | 0.94 | 0.78-1.13 | 0.30 | 0.85 | 0.65-1.12 | 0.86 | 0.85 | 0.66-1.09 | 0.89 |
| 3 | 0.92 | 0.74-1.43 | 0.30 | 1.01 | 0.77-1.31 | 0.13 | 1.23 | 0.94-1.61 | **0.004** |
| 4 | 0.90 | 0.71-1.15 | 0.88 | 0.88 | 0.62-1.24 | 0.97 | 1.10 | 0.72-1.69 | 0.32 |
| *Era 2* | | | | | | | | | |
| 1 | 1 | ref | ref | 1 | ref | ref | 1 | ref | ref |
| 2 | 0.83 | 0.60-1.14 | 0.50 | 0.63 | 0.38-1.04 | 0.11 | 0.95 | 0.60-1.52 | 0.95 |
| 3 | 0.84 | 0.61-1.16 | 0.68 | 0.64 | 0.40-1.03 | 0.44 | 1.20 | 0.76-1.90 | 0.05 |
| 4 | 0.99 | 0.72-1.36 | 0.19 | 0.83 | 0.54-1.28 | 0.75 | 1.40 | 0.89-2.20 | **0.007** |
| **ATxV Quintiles** | | | | | | | | | |
| *Era 1* | | | | | | | | | |
| 1 | 1 | ref | ref | 1 | ref | ref | 1 | ref | ref |
| 2 | 0.90 | 0.73-1.11 | 0.37 | 0.98 | 0.75-1.27 | 0.19 | 0.94 | 0.75-1.20 | 0.30 |
| 3 | 0.97 | 0.77-1.22 | 0.18 | 0.95 | 0.70-1.30 | 0.21 | 0.84 | 0.60-1.16 | 0.76 |
| 4 | 0.92 | 0.73-1.17 | 0.16 | 1.03 | 0.74-1.44 | 0.10 | 1.30 | 0.91-1.85 | **0.004** |
| 5 | 0.88 | 0.65-1.19 | 0.82 | 0.91 | 0.62-1.34 | 0.68 | 0.90 | 0.55-1.48 | 0.80 |
| *Era 2* | | | | | | | | | |
| 1 | 1 | ref | ref | 1 | ref | ref | 1 | ref | ref |
| 2 | 1.02 | 0.64-1.61 | 0.29 | 0.90 | 0.52-1.53 | 0.56 | 0.83 | 0.50-1.39 | 0.71 |
| 3 | 0.74 | 0.48-1.12 | 0.90 | 0.57 | 0.29-1.13 | 0.52 | 1.14 | 0.66-1.98 | 0.07 |
| 4 | 1.02 | 0.65-1.58 | 0.06 | 0.59 | 0.32-1.08 | 0.77 | 1.42 | 0.87-2.32 | **0.001** |
| 5 | 1.04 | 0.68-1.58 | 0.06 | 0.87 | 0.51-1.50 | 0.38 | 1.32 | 0.81-2.17 | **0.004** |

^a^ Multivariable models adjusted for characteristics at listing including etiology of liver disease, sex, race/ethnicity, public insurance, diabetes, dialysis, BMI, Child Pugh class, MELD, region, age, and blood type. **^b^** Effect sizes are sub-hazard ratios. **^c^** Etiology by ATxV interaction p-values (vs ALD)

Supplementary Table 14: Multivariable^a^ Sub-Hazard Ratios For Probability Of Liver Transplantation By Indication And Listing Era in Quartiles and Quintiles

| **ATxV** | **ALD** | | **HCC** | | | **Other** | | |
| --- | --- | --- | --- | --- | --- | --- | --- | --- |
|  | **sHR^b^** | **95% CI** | **sHR^b^** | **95% CI** | **P value** | **sHR^b^** | **95% CI** | **P value** |
|  |  |  |  |  | **vs ALD^c^** |  |  | **vs ALD^c^** |
| **ATxV quartiles** | | | | | | | | |
| *Era 1* | | | | | | | | |
| 1 | 1 | ref | 1 | ref | ref | 1 | ref | ref |
| 2 | 1.23 | 1.05-1.43 | 1.02 | 0.92-1.14 | **0.008** | 1.13 | 0.98-1.30 | 0.14 |
| 3 | 1.22 | 1.04-1.44 | 0.99 | 0.84-1.17 | **0.005** | 0.97 | 0.82-1.14 | **0.001** |
| 4 | 1.35 | 1.12-1.63 | 1.03 | 0.88-1.21 | **0.001** | 1.14 | 0.92-1.41 | 0.07 |
| *Era 2* | | | | | | | | |
| 1 | 1 | ref | 1 | ref | ref | 1 | ref | ref |
| 2 | 1.13 | 0.87-1.46 | 1.11 | 0.93-1.33 | 0.90 | 1.04 | 0.84-1.29 | 0.56 |
| 3 | 1.22 | 0.90-1.65 | 1.13 | 0.88-1.45 | 0.48 | 1.02 | 0.81-1.29 | 0.16 |
| 4 | 1.19 | 0.90-1.57 | 0.96 | 0.76-1.21 | 0.05 | 0.90 | 0.71-1.15 | **0.04** |
| **ATxV quintiles** | | | | | | | | |
| *Era 1* | | | | | | | | |
| 1 | 1 | ref | 1 | ref | ref | 1 | ref | ref |
| 2 | 1.22 | 1.04-1.43 | 0.98 | 0.89-1.08 | **0.004** | 1.10 | 0.95-1.28 | 0.09 |
| 3 | 1.19 | 0.99-1.42 | 0.99 | 0.84-1.15 | **0.01** | 1.01 | 0.86-1.19 | **0.01** |
| 4 | 1.32 | 1.10-1.59 | 0.98 | 0.85-1.14 | **<0.001** | 0.98 | 0.79-1.21 | **<0.001** |
| 5 | 1.41 | 1.14-1.74 | 1.03 | 0.85-1.25 | **0.002** | 1.16 | 0.91-1.49 | 0.09 |
| *Era 2* | | | | | | | | |
| 1 | 1 | ref | 1 | ref | ref | 1 | ref | ref |
| 2 | 1.36 | 0.95-1.95 | 1.21 | 0.95-1.54 | 0.43 | 1.01 | 0.78-1.31 | **0.046** |
| 3 | 1.50 | 1.05-2.15 | 1.41 | 1.08-1.84 | 0.61 | 1.15 | 0.87-1.51 | **0.03** |
| 4 | 1.35 | 0.95-1.92 | 1.15 | 0.87-1.52 | 0.22 | 0.89 | 0.71-1.13 | **0.002** |
| 5 | 1.35 | 0.94-1.92 | 0.99 | 0.74-1.33 | **0.02** | 0.88 | 0.69-1.13 | **<0.001** |

^a^ Multivariable models adjusted for characteristics at listing including etiology of liver disease, sex, race/ethnicity, public insurance, diabetes, dialysis, BMI, Child Pugh class, MELD, region, age, and blood type. **^b^** Effect sizes are sub-hazard ratios. **^c^** Etiology by ATxV interaction p-values (vs ALD) ^d^ Metabolic steatohepatitis

Supplementary Table 15: Multivariable^a^ sub-Hazard Ratios for Probability of Liver Transplantation by Other Indication Type and Listing Era in Quartiles and Quintiles

| **ATxV** | **MASH^d^** | | | **Viral hepatitis** | | | **Immune** | | |
| --- | --- | --- | --- | --- | --- | --- | --- | --- | --- |
|  | **sHR^b^** | **95% CI** | **P value**  **vs ALD^c^** | **sHR^b^** | **95% CI** | **P value** | **sHR^b^** | **95% CI** | **P value** |
|  |  |  |  |  |  | **vs ALD^c^** |  |  | **vs ALD^c^** |
| **ATxV Quartiles** | | | | | | | | | |
| *Era 1* | | | | | | | | | |
| 1 | 1 | ref | ref | 1 | ref | ref | 1 | ref | ref |
| 2 | 1.06 | 0.91-1.24 | **0.04** | 1.20 | 0.96-1.51 | 0.86 | 1.22 | 0.99-1.49 | 0.93 |
| 3 | 1.00 | 0.82-1.22 | **0.03** | 0.94 | 0.72-1.23 | **0.03** | 0.96 | 0.75-1.23 | **0.04** |
| 4 | 1.10 | 0.87-1.38 | 0.08 | 1.21 | 0.87-1.69 | 0.46 | 1.11 | 0.77-1.60 | 0.28 |
| *Era 2* | | | | | | | | | |
| 1 | 1 | ref | ref | 1 | ref | ref | 1 | ref | ref |
| 2 | 0.98 | 0.81-1.18 | 0.25 | 1.21 | 0.74-1.99 | 0.77 | 0.96 | 0.74-1.26 | 0.29 |
| 3 | 0.94 | 0.75-1.18 | **0.02** | 1.10 | 0.72-1.68 | 0.60 | 0.95 | 0.71-1.26 | 0.06 |
| 4 | 0.87 | 0.70-1.08 | **0.009** | 1.00 | 0.66-1.52 | 0.40 | 0.76 | 0.58-0.99 | **0.001** |
| **ATxV Quintiles** | | | | | | | | | |
| *Era 1* | | | | | | | | | |
| 1 | 1 | ref | ref | 1 | ref | ref | 1 | ref | ref |
| 2 | 1.02 | 0.85-1.22 | **0.02** | 1.27 | 1.02-1.58 | 0.72 | 1.13 | 0.92-1.38 | 0.44 |
| 3 | 0.98 | 0.82-1.17 | **0.02** | 1.07 | 0.82-1.38 | 0.32 | 1.09 | 0.84-1.42 | 0.51 |
| 4 | 0.97 | 0.76-1.24 | **0.004** | 1.00 | 0.73-1.36 | 0.05 | 0.99 | 0.71-1.40 | 0.07 |
| 5 | 1.09 | 0.84-1.41 | 0.07 | 1.28 | 0.85-1.95 | 0.62 | 1.24 | 0.84-1.83 | 0.51 |
| *Era 2* | | | | | | | | | |
| 1 | 1 | ref | ref | 1 | ref | ref | 1 | ref | ref |
| 2 | 1.04 | 0.82-1.34 | 0.07 | 1.17 | 0.65-2.10 | 0.58 | 1.11 | 0.82-1.48 | 0.25 |
| 3 | 1.15 | 0.86-1.55 | **0.03** | 1.31 | 0.72-2.40 | 0.62 | 1.13 | 0.80-1.60 | 0.09 |
| 4 | 0.91 | 0.69-1.20 | **0.005** | 1.02 | 0.60-1.73 | 0.28 | 0.83 | 0.63-1.10 | **0.006** |
| 5 | 0.88 | 0.67-1.17 | **0.001** | 1.02 | 0.60-1.75 | 0.29 | 0.83 | 0.62-1.12 | **0.004** |

^a^ Multivariable models adjusted for characteristics at listing including etiology of liver disease, sex, race/ethnicity, public insurance, diabetes, dialysis, BMI, Child Pugh class, MELD, region, age, and blood type. **^b^** Effect sizes are sub-hazard ratios. **^c^** Etiology by ATxV interaction p-values (vs ALD) ^d^ Metabolic steatohepatitis
